# Supplementary material for: Developmental atlas of phase-amplitude coupling between physiologic high-frequency oscillations and slow waves
Source: Nat Commun. 2023 Oct 13;14:6435. doi: 10.1038/s41467-023-42091-y (PMC10575956; doi:10.1038/s41467-023-42091-y)
Supplement: Supplementary file 1 — Supplementary Information [file 41467_2023_42091_MOESM1_ESM.pdf]

Developmental atlas of phase-amplitude coupling between physiologic high-frequency oscillation and slow waves

Kazuki Sakakura ; Naoto Kuroda ; Masaki Sonoda ; Takumi Mitsuhashi ;
Ethan Firestone ; Aimee F. Luat ; Neena I. Marupudi ; Sandeep Sood ;
Eishi Asano

This supplementary document includes the following information.

Supplementary Tables 1 - 41.

Supplementary Figures 1 – 6.

Age (in years)	Number of patients from the left hemisphere	Number of patients from the right hemisphere	Number of patients from both hemispheres
1	3	4	0
2	0	4	0
3	2	1	0
4	2	2	0
5	4	2	1
6	0	1	2
7	1	1	0
8	4	3	1
9	3	0	2
10	3	7	0
11	6	4	0
12	4	2	0
13	1	4	0
14	5	3	1
15	3	3	2
16	3	2	1
17	6	3	1
18	0	0	1
19	0	3	0
20-	2	0	1
Total	52	49	13

Supplementary Table 1. Distribution of patient ages. A total of 114 patients were analyzed in the present study. Three patients were aged 21 years or older.

Lobe	Slope (/√year)	Uncorrected two-sided p-value	t-value	DF	Lower 95% CI	Upper 95% CI
Frontal	5.8×10^{-3}	$*6.9 \times 10^{-4}$	3.4	2895	2.5×10^{-3}	9.2×10^{-3}
Temporal	5.1×10^{-3}	$*3.0 \times 10^{-4}$	3.6	2314	2.4×10^{-3}	7.9×10^{-3}
Parietal	8.2×10^{-3}	$*3.0 \times 10^{-5}$	4.2	1949	4.4×10^{-3}	0.012
Occipital	0.049	$*4.1 \times 10^{-11}$	6.7	831	0.034	0.063

Supplementary Table 2. The results of ancillary regression analysis to assess the effect of √age on $MI_{\geq 80\ Hz\ \&\ 0.5\text{-}1\ Hz}$ in a given brain lobe. Here, we present the results of ancillary analyses, excluding three patients of 21 years old and above. $MI_{\geq 80\ Hz\ and\ 0.5\text{-}1\ Hz}$ denotes the strength of coupling between the amplitude of high-frequency $oscillation_{\geq 80\ Hz}$ and the phase of slow-$wave_{0.5\text{-}1\ Hz}$. CI: confidence interval. DF: degree of freedom. *: significant with False Discovery Rate (FDR) correction on the regression analysis.

Lobe	Slope (/√year)	Uncorrected two-sided p-value	t-value	DF	Lower 95% CI	Upper 95% CI
Frontal	-0.45	$*2.7 \times 10^{-49}$	-15.0	2895	-0.51	-0.39
Temporal	-0.38	$*1.6 \times 10^{-34}$	-12.5	2314	-0.44	-0.32
Parietal	-0.39	$*4.9 \times 10^{-25}$	-10.5	1949	-0.46	-0.32
Occipital	0.13	0.15	1.4	831	-0.046	0.30

Supplementary Table 3. The results of ancillary regression analysis to assess the effect of √age on $HFO_{HIL \geq 80\ Hz}$ occurrence rate in a given brain lobe. Here, we present the results of ancillary analyses, excluding three patients of 21 years old and above. $HFO_{HIL \geq 80\ Hz}$: high-frequency $oscillation_{\geq 80\ Hz}$ defined by the Hilbert method. CI: confidence interval. DF: degree of freedom. *: significant with False Discovery Rate (FDR) correction on the regression analysis.

Parameters	Estimate	SE	t-value	DF	Uncorrected two-sided p-value	Lower 95% CI	Upper 95% CI
Intercept	0.15	0.026	5.80	2973	7.3×10^{-9}	0.099	0.20
Age (√year)	0.0041	0.0056	0.74	2973	0.46	-0.0068	0.015
Number of ASMs	-0.014	0.0062	-2.20	2973	0.028	-0.026	-0.0015
MRI lesion (yes = 1)	-0.019	0.011	-1.73	2973	0.083	-0.041	0.0025
Side (left = 1)	0.0056	0.010	0.54	2973	0.59	-0.015	0.027
SOZ (frontal = 1)	0.018	0.012	1.44	2973	0.15	-0.0064	0.042
Sex (female = 1)	0.019	0.010	1.80	2973	0.073	-0.0017	0.039

Supplementary Table 4. The results of mixed model analysis to assess the independent effect of √age on modulation index (MI)$_{\geq 80\ Hz\ \&\ 0.5\text{-}1\ Hz}$ in the frontal lobe. ASMs: antiseizure medications. CI: confidence interval. DF: degree of freedom. MI: modulation index. SE: standard error. SOZ: seizure onset zone.

Parameters	Estimate	SE	t-value	DF	Uncorrected two-sided p-value	Lower 95% CI	Upper 95% CI
Intercept	0.085	0.018	4.61	2388	4.3×10^{-6}	0.049	0.12
Age (√year)	0.0090	0.0038	2.37	2388	0.018	0.0015	0.017
Number of ASMs	-0.0058	0.0043	-1.37	2388	0.17	-0.014	0.0025
MRI lesion (yes = 1)	0.0041	0.0078	0.53	2388	0.60	-0.011	0.019
Side (left = 1)	0.0055	0.0073	0.75	2388	0.45	-0.0088	0.020
SOZ (temporal = 1)	0.0020	0.0071	0.28	2388	0.78	-0.012	0.016
Sex (female = 1)	0.011	0.0072	1.56	2388	0.12	-0.0029	0.025

Supplementary Table 5. The results of mixed model analysis to assess the independent effect of √age on modulation index (MI)$_{\geq 80\ Hz\ \&\ 0.5\text{-}1\ Hz}$ in the temporal lobe. ASMs: antiseizure medications. CI: confidence interval. DF: degree of freedom. MI: modulation index. SE: standard error. SOZ: seizure onset zone.

Parameters	Estimate	SE	t-value	DF	Uncorrected two-sided p-value	Lower 95% CI	Upper 95% CI
Intercept	0.12	0.024	5.18	1994	2.4×10^{-7}	0.078	0.17
Age (√year)	0.0065	0.0051	1.27	1994	0.20	-0.0035	0.016
Number of ASMs	-0.0031	0.0058	-0.54	1994	0.59	-0.014	0.0082
MRI lesion (yes = 1)	-0.013	0.011	-1.26	1994	0.21	-0.034	0.0074
Side (left = 1)	0.0034	0.0097	0.36	1994	0.72	-0.016	0.022
SOZ (parietal = 1)	-0.0075	0.011	-0.68	1994	0.50	-0.029	0.014
Sex (female = 1)	0.020	0.0097	2.04	1994	0.041	8.0×10^{-4}	0.039

Supplementary Table 6. The results of mixed model analysis to assess the independent effect of √age on modulation index (MI)$_{\geq 80\ Hz\ \&\ 0.5\text{-}1\ Hz}$ in the parietal lobe. ASMs: antiseizure medications. CI: confidence interval. DF: degree of freedom. MI: modulation index. SE: standard error. SOZ: seizure onset zone.

Parameters	Estimate	SE	t-value	DF	Uncorrected two-sided p-value	Lower 95% CI	Upper 95% CI
Intercept	0.14	0.066	2.11	868	0.036	0.0094	0.27
Age (√year)	0.046	0.014	3.39	868	7.4×10^{-4}	0.019	0.072
Number of ASMs	0.0018	0.016	0.11	868	0.91	-0.029	0.033
MRI lesion (yes = 1)	0.0057	0.028	0.20	868	0.84	-0.050	0.061
Side (left = 1)	-0.052	0.027	-1.96	868	0.050	-0.10	4.3×10^{-5}
SOZ (occipital = 1)	0.015	0.034	0.45	868	0.65	-0.052	0.083
Sex (female = 1)	0.022	0.026	0.86	868	0.39	-0.029	0.074

Supplementary Table 7. The results of mixed model analysis to assess the independent effect of √age on modulation index (MI)$_{\geq 80\ Hz\ \&\ 0.5\text{-}1\ Hz}$ in the occipital lobe. ASMs: antiseizure medications. CI: confidence interval. DF: degree of freedom. MI: modulation index. SE: standard error. SOZ: seizure onset zone.

Parameters	Estimate	SE	t-value	DF	Uncorrected two-sided p-value	Lower 95% CI	Upper 95% CI
Intercept	0.15	0.029	5.65	2890	1.7×10^{-8}	0.099	0.20
Age (√year)	0.0032	0.0060	0.53	2890	0.60	-0.0086	0.015
Number of ASMs	-0.014	0.0062	-2.29	2890	0.022	-0.026	-0.0020
MRI lesion (yes = 1)	-0.019	0.011	-1.67	2890	0.095	-0.041	0.0032
Side (left = 1)	0.0070	0.011	0.67	2890	0.51	-0.014	0.028
SOZ (frontal = 1)	0.018	0.012	1.44	2890	0.15	-0.0064	0.042
Sex (female = 1)	0.020	0.011	1.89	2890	0.059	-7.5×10^{-4}	0.040

Supplementary Table 8. The results of ancillary mixed model analysis to assess the independent effect of √age on modulation index $(MI)_{\geq 80\ Hz\ \&\ 0.5\text{-}1\ Hz}$ in the frontal lobe. Here, we present the results of ancillary analysis, excluding three patients of 21 years old and above. ASMs: antiseizure medications. CI: confidence interval. DF: degree of freedom. MI: modulation index. SE: standard error. SOZ: seizure onset zone.

Parameters	Estimate	SE	t-value	DF	Uncorrected two-sided p-value	Lower 95% CI	Upper 95% CI
Intercept	0.091	0.019	4.86	2309	1.3×10^{-6}	0.054	0.13
Age (√year)	0.0068	0.0041	1.66	2309	0.096	-0.0012	0.015
Number of ASMs	-0.0052	0.0042	-1.25	2309	0.21	-0.013	0.0030
MRI lesion (yes = 1)	0.0031	0.0077	0.41	2309	0.68	-0.012	0.018
Side (left = 1)	0.0045	0.0072	0.62	2309	0.53	-0.0097	0.019
SOZ (temporal = 1)	0.0033	0.0070	0.47	2309	0.63	-0.010	0.017
Sex (female = 1)	0.0098	0.0071	1.37	2309	0.17	-0.0042	0.024

Supplementary Table 9. The results of ancillary mixed model analysis to assess the independent effect of √age on modulation index $(MI)_{\geq 80\ Hz\ \&\ 0.5\text{-}1\ Hz}$ in the temporal lobe. Here, we present the results of ancillary analysis, excluding three patients of 21 years old and above. ASMs: antiseizure medications. CI: confidence interval. DF: degree of freedom. MI: modulation index. SE: standard error. SOZ: seizure onset zone.

Parameters	Estimate	SE	t-value	DF	Uncorrected two-sided p-value	Lower 95% CI	Upper 95% CI
Intercept	0.13	0.025	5.32	1944	1.2×10^{-7}	0.084	0.18
Age (√year)	0.0036	0.0055	0.65	1944	0.52	-0.0072	0.014
Number of ASMs	-0.0038	0.0058	-0.65	1944	0.51	-0.015	0.0076
MRI lesion (yes = 1)	-0.013	0.011	-1.21	1944	0.23	-0.033	0.0079
Side (left = 1)	0.0040	0.0098	0.41	1944	0.68	-0.015	0.023
SOZ (parietal = 1)	-0.0063	0.011	-0.57	1944	0.57	-0.028	0.015
Sex (female = 1)	0.020	0.0099	2.02	1944	0.044	5.7×10^{-4}	0.039

Supplementary Table 10. The results of ancillary mixed model analysis to assess the independent effect of √age on modulation index (MI)$_{\geq 80\ Hz\ \&\ 0.5\text{-}1\ Hz}$ in the parietal lobe. Here, we present the results of ancillary analysis, excluding three patients of 21 years old and above. ASMs: antiseizure medications. CI: confidence interval. DF: degree of freedom. MI: modulation index. SE: standard error. SOZ: seizure onset zone.

Parameters	Estimate	SE	t-value	DF	Uncorrected two-sided p-value	Lower 95% CI	Upper 95% CI
Intercept	0.14	0.067	2.03	826	0.042	0.0048	0.27
Age (√year)	0.044	0.014	3.07	826	0.0022	0.016	0.073
Number of ASMs	-0.0035	0.016	-0.22	826	0.83	-0.035	0.028
MRI lesion (yes = 1)	0.010	0.028	0.37	826	0.71	-0.045	0.036
Side (left = 1)	-0.041	0.026	-1.53	826	0.13	-0.092	0.011
SOZ (occipital = 1)	0.028	0.035	0.80	826	0.43	-0.041	0.097
Sex (female = 1)	0.033	0.026	1.26	826	0.21	-0.018	0.084

Supplementary Table 11. The results of ancillary mixed model analysis to assess the independent effect of √age on modulation index (MI)$_{\geq 80\ Hz\ \&\ 0.5\text{-}1\ Hz}$ in the occipital lobe. Here, we present the results of ancillary analysis, excluding three patients of 21 years old and above. ASMs: antiseizure medications. CI: confidence interval. DF: degree of freedom. MI: modulation index. SE: standard error. SOZ: seizure onset zone.

Parameters	Estimate	SE	t-value	DF	Uncorrected two-sided p-value	Lower 95% CI	Upper 95% CI
Intercept	8.69	19.03	0.46	9	0.66	-34.38	51.76
Age (√year)	-0.62	1.40	-0.44	9	0.67	-3.79	2.55
Number of ASMs	-1.71	3.75	-0.46	9	0.66	-10.21	6.78
MRI lesion (yes = 1)	0.24	0.54	0.44	9	0.67	-0.98	1.45
Side (left = 1)	-4.10	9.29	-0.44	9	0.67	-25.11	16.92
SOZ (frontal = 1)	2.06	4.64	0.44	9	0.67	-8.43	12.56
Sex (female = 1)	-3.06	6.72	-0.45	9	0.66	-18.27	12.16

Supplementary Table 12. The results of ancillary mixed model analysis to assess the independent effect of √age on modulation index (MI)$_{\geq 80\ \mathrm{Hz}\ \&\ 0.5\text{-}1\ \mathrm{Hz}}$ in the anterior cingulate gyrus. ASMs: antiseizure medications. CI: confidence interval. DF: degree of freedom. MI: modulation index. SE: standard error. SOZ: seizure onset zone. **Supplementary Figure 4** shows the spatial extent of a given region of interest.

Parameters	Estimate	SE	t-value	DF	Uncorrected two-sided p-value	Lower 95% CI	Upper 95% CI
Intercept	0.14	0.035	3.96	489	8.6×10^{-5}	0.070	0.21
Age (√year)	0.0041	0.0077	0.53	489	0.60	-0.011	0.019
Number of ASMs	-0.0095	0.0086	-1.11	489	0.27	-0.026	0.0073
MRI lesion (yes = 1)	-0.020	0.015	-1.35	489	0.18	-0.049	0.0090
Side (left = 1)	0.0076	0.014	0.55	489	0.58	-0.020	0.035
SOZ (frontal = 1)	0.015	0.017	0.88	489	0.38	-0.019	0.049
Sex (female = 1)	0.021	0.014	1.53	489	0.13	-0.0060	0.048

Supplementary Table 13. The results of ancillary mixed model analysis to assess the independent effect of √age on modulation index (MI)$_{\geq 80\ \mathrm{Hz}\ \&\ 0.5\text{-}1\ \mathrm{Hz}}$ in the anterior middle frontal gyrus. ASMs: antiseizure medications. CI: confidence interval. DF: degree of freedom. MI: modulation index. SE: standard error. SOZ: seizure onset zone. **Supplementary Figure 4** shows the spatial extent of a given region of interest.

Parameters	Estimate	SE	t-value	DF	Uncorrected two-sided p-value	Lower 95% CI	Upper 95% CI
Intercept	0.091	0.034	2.72	441	0.0068	0.025	0.16
Age (√year)	0.015	0.0067	2.20	441	0.029	0.0015	0.028
Number of ASMs	-0.0046	0.0079	-0.58	441	0.56	-0.020	0.011
MRI lesion (yes = 1)	-0.0021	0.014	-0.15	441	0.88	-0.029	0.025
Side (left = 1)	-2.1×10^{-4}	0.013	-0.016	441	0.99	-0.026	0.025
SOZ (temporal = 1)	0.012	0.012	0.97	441	0.33	-0.012	0.036
Sex (female = 1)	0.0059	0.013	0.47	441	0.64	-0.019	0.031

Supplementary Table 14. The results of ancillary mixed model analysis to assess the independent effect of √age on modulation index (MI)$_{\geq 80\ Hz\ \&\ 0.5\text{-}1\ Hz}$ in the fusiform gyrus. ASMs: antiseizure medications. CI: confidence interval. DF: degree of freedom. MI: modulation index. SE: standard error. SOZ: seizure onset zone. **Supplementary Figure 4** shows the spatial extent of a given region of interest. The observed √age effect did not withstand the false discovery rate correction that was applied for the number of regions of interest.

Parameters	Estimate	SE	t-value	DF	Uncorrected two-sided p-value	Lower 95% CI	Upper 95% CI
Intercept	0.13	0.042	3.04	258	0.0026	0.045	0.21
Age (√year)	0.012	0.0085	1.41	258	0.16	-0.0047	0.029
Number of ASMs	-0.0076	0.010	-0.74	258	0.46	-0.028	0.013
MRI lesion (yes = 1)	-0.0031	0.018	-0.17	258	0.86	-0.039	0.033
Side (left = 1)	-0.0062	0.017	-0.36	258	0.72	-0.040	0.027
SOZ (parietal = 1)	-0.034	0.018	-1.91	258	0.057	-0.068	0.0011
Sex (female = 1)	0.016	0.017	0.96	258	0.34	-0.017	0.050

Supplementary Table 15. The results of ancillary mixed model analysis to assess the independent effect of √age on modulation index (MI)$_{\geq 80\ Hz\ \&\ 0.5\text{-}1\ Hz}$ in the inferior parietal lobule. ASMs: antiseizure medications. CI: confidence interval. DF: degree of freedom. MI: modulation index. SE: standard error. SOZ: seizure onset zone. **Supplementary Figure 4** shows the spatial extent of a given region of interest.

Parameters	Estimate	SE	t-value	DF	Uncorrected two-sided p-value	Lower 95% CI	Upper 95% CI
Intercept	0.092	0.025	3.73	394	2.2×10^{-4}	0.044	0.14
Age (√year)	0.0068	0.0051	1.32	394	0.19	-0.0033	0.017
Number of ASMs	-0.0052	0.0055	-0.95	394	0.34	-0.016	0.0056
MRI lesion (yes = 1)	0.0028	0.011	0.26	394	0.79	-0.018	0.024
Side (left = 1)	0.010	0.0099	1.05	394	0.30	-0.0091	0.030
SOZ (temporal = 1)	-2.8×10^{-4}	0.0098	-0.029	394	0.98	-0.020	0.019
Sex (female = 1)	0.018	0.0099	1.83	394	0.069	-0.0014	0.037

Supplementary Table 16. The results of ancillary mixed model analysis to assess the independent effect of √age on modulation index $(MI)_{\geq 80\ Hz\ \&\ 0.5\text{-}1\ Hz}$ in the inferior temporal gyrus. ASMs: antiseizure medications. CI: confidence interval. DF: degree of freedom. MI: modulation index. SE: standard error. SOZ: seizure onset zone. **Supplementary Figure 4** shows the spatial extent of a given region of interest.

Parameters	Estimate	SE	t-value	DF	Uncorrected two-sided p-value	Lower 95% CI	Upper 95% CI
Intercept	0.17	0.070	2.47	508	0.014	0.036	0.31
Age (√year)	0.042	0.014	3.01	508	0.0027	0.015	0.069
Number of ASMs	0.0035	0.017	0.21	508	0.84	-0.030	0.037
MRI lesion (yes = 1)	-0.0065	0.030	-0.22	508	0.83	-0.065	0.052
Side (left = 1)	-0.067	0.028	-2.41	508	0.016	-0.12	-0.012
SOZ (occipital = 1)	0.022	0.036	0.60	508	0.55	-0.050	0.093
Sex (female = 1)	0.011	0.027	0.39	508	0.70	-0.043	0.064

Supplementary Table 17. The results of ancillary mixed model analysis to assess the independent effect of √age on modulation index $(MI)_{\geq 80\ Hz\ \&\ 0.5\text{-}1\ Hz}$ in the lateral occipital gyrus. ASMs: antiseizure medications. CI: confidence interval. DF: degree of freedom. MI: modulation index. SE: standard error. SOZ: seizure onset zone. **Supplementary Figure 4** shows the spatial extent of a given region of interest. The observed √age effect withstood the false discovery rate correction that was applied for the number of regions of interest.

Parameters	Estimate	SE	t-value	DF	Uncorrected two-sided p-value	Lower 95% CI	Upper 95% CI
Intercept	0.083	0.086	0.98	353	0.33	-0.085	0.25
Age (√year)	0.057	0.018	3.15	353	0.0018	0.021	0.093
Number of ASMs	2.8×10^{-4}	0.021	0.013	353	0.99	-0.040	0.041
MRI lesion (yes = 1)	0.0080	0.037	0.21	353	0.83	-0.065	0.081
Side (left = 1)	-0.033	0.035	-0.95	353	0.34	-0.10	0.036
SOZ (occipital = 1)	0.012	0.042	0.28	353	0.78	-0.071	0.095
Sex (female = 1)	0.034	0.034	1.00	353	0.32	-0.033	0.10

Supplementary Table 18. The results of ancillary mixed model analysis to assess the independent effect of √age on modulation index (MI)$_{\geq 80\ \mathrm{Hz}\ \&\ 0.5\text{-}1\ \mathrm{Hz}}$ in the medial occipital gyrus. ASMs: antiseizure medications. CI: confidence interval. DF: degree of freedom. MI: modulation index. SE: standard error. SOZ: seizure onset zone. **Supplementary Figure 4** shows the spatial extent of a given region of interest. The observed √age effect withstood the false discovery rate correction that was applied for the number of regions of interest.

Parameters	Estimate	SE	t-value	DF	Uncorrected two-sided p-value	Lower 95% CI	Upper 95% CI
Intercept	0.079	0.031	2.59	190	0.010	0.019	0.14
Age (√year)	0.012	0.0068	1.71	190	0.086	-0.0018	0.025
Number of ASMs	-0.0097	0.0068	-1.41	190	0.16	-0.023	0.0038
MRI lesion (yes = 1)	0.0038	0.014	0.28	190	0.78	-0.023	0.031
Side (left = 1)	0.011	0.013	0.87	190	0.39	-0.014	0.037
SOZ (temporal = 1)	0.0074	0.014	0.51	190	0.61	-0.021	0.036
Sex (female = 1)	-7.9×10^{-4}	0.013	-0.063	190	0.95	-0.026	0.024

Supplementary Table 19. The results of ancillary mixed model analysis to assess the independent effect of √age on modulation index (MI)$_{\geq 80\ \mathrm{Hz}\ \&\ 0.5\text{-}1\ \mathrm{Hz}}$ in the medial temporal region. ASMs: antiseizure medications. CI: confidence interval. DF: degree of freedom. MI: modulation index. SE: standard error. SOZ: seizure onset zone. **Supplementary Figure 4** shows the spatial extent of a given region of interest.

Parameters	Estimate	SE	t-value	DF	Uncorrected two-sided p-value	Lower 95% CI	Upper 95% CI
Intercept	0.074	0.021	3.57	472	3.9×10^{-4}	0.033	0.11
Age (√year)	0.013	0.0043	2.91	472	0.0038	0.0040	0.021
Number of ASMs	-0.0059	0.0051	-1.17	472	0.24	-0.016	0.0041
MRI lesion (yes = 1)	0.0085	0.0091	0.94	472	0.35	-0.0094	0.026
Side (left = 1)	0.0019	0.0085	0.23	472	0.82	-0.015	0.019
SOZ (temporal = 1)	-0.0066	0.0084	-0.78	472	0.44	-0.023	0.010
Sex (female = 1)	0.019	0.0083	2.30	472	0.022	0.0028	0.053

Supplementary Table 20. The results of ancillary mixed model analysis to assess the independent effect of √age on modulation index (MI)$_{\geq 80\ Hz\ \&\ 0.5\text{-}1\ Hz}$ in the middle temporal gyrus. ASMs: antiseizure medications. CI: confidence interval. DF: degree of freedom. MI: modulation index. SE: standard error. SOZ: seizure onset zone. **Supplementary Figure 4** shows the spatial extent of a given region of interest. The observed √age effect withstood the false discovery rate correction that was applied for the number of regions of interest.

Parameters	Estimate	SE	t-value	DF	Uncorrected two-sided p-value	Lower 95% CI	Upper 95% CI
Intercept	0.15	0.048	3.10	334	0.0021	0.054	0.24
Age (√year)	0.012	0.0093	1.24	334	0.22	-0.0068	0.030
Number of ASMs	-0.023	0.011	-2.01	334	0.045	-0.045	-5.1×10^{-4}
MRI lesion (yes = 1)	-0.015	0.018	-0.85	334	0.40	-0.051	0.020
Side (left = 1)	-0.010	0.017	-0.62	334	0.54	-0.043	0.022
SOZ (frontal = 1)	0.0033	0.019	0.17	334	0.86	-0.035	0.041
Sex (female = 1)	0.030	0.017	1.82	334	0.069	-0.0023	0.063

Supplementary Table 21. The results of ancillary mixed model analysis to assess the independent effect of √age on modulation index (MI)$_{\geq 80\ Hz\ \&\ 0.5\text{-}1\ Hz}$ in the orbitofrontal gyrus. ASMs: antiseizure medications. CI: confidence interval. DF: degree of freedom. MI: modulation index. SE: standard error. SOZ: seizure onset zone. **Supplementary Figure 4** shows the spatial extent of a given region of interest.

Parameters	Estimate	SE	t-value	DF	Uncorrected two-sided p-value	Lower 95% CI	Upper 95% CI
Intercept	0.12	0.065	1.87	77	0.065	-0.0079	0.25
Age (√year)	0.017	0.013	1.25	77	0.22	-0.0099	0.043
Number of ASMs	2.6×10^{-4}	0.019	0.014	77	0.99	-0.037	0.037
MRI lesion (yes = 1)	-0.011	0.031	-0.36	77	0.72	-0.073	0.051
Side (left = 1)	-0.016	0.033	-0.49	77	0.63	-0.082	0.050
SOZ (frontal = 1)	0.027	0.028	0.93	77	0.35	-0.030	0.083
Sex (female = 1)	-0.018	0.028	-0.64	77	0.53	-0.074	0.038

Supplementary Table 22. The results of ancillary mixed model analysis to assess the independent effect of √age on modulation index (MI)$_{\geq 80\text{ Hz} \,\&\, 0.5\text{-}1\text{ Hz}}$ in the paracentral lobule. ASMs: antiseizure medications. CI: confidence interval. DF: degree of freedom. MI: modulation index. SE: standard error. SOZ: seizure onset zone. **Supplementary Figure 4** shows the spatial extent of a given region of interest.

Parameters	Estimate	SE	t-value	DF	Uncorrected two-sided p-value	Lower 95% CI	Upper 95% CI
Intercept	0.15	0.029	5.08	742	4.9×10^{-7}	0.091	0.21
Age (√year)	7.3×10^{-4}	0.0060	0.12	742	0.90	-0.011	0.013
Number of ASMs	-0.0012	0.0072	-0.16	742	0.87	-0.015	0.013
MRI lesion (yes = 1)	-0.020	0.012	-1.59	742	0.11	-0.044	0.0047
Side (left = 1)	0.0050	0.011	0.44	742	0.66	-0.017	0.027
SOZ (parietal = 1)	-0.012	0.013	-0.95	742	0.34	-0.038	0.013
Sex (female = 1)	0.014	0.011	1.26	742	0.21	-0.0079	0.036

Supplementary Table 23. The results of ancillary mixed model analysis to assess the independent effect of √age on modulation index (MI)$_{\geq 80\text{ Hz} \,\&\, 0.5\text{-}1\text{ Hz}}$ in the postcentral gyrus. ASMs: antiseizure medications. CI: confidence interval. DF: degree of freedom. MI: modulation index. SE: standard error. SOZ: seizure onset zone. **Supplementary Figure 4** shows the spatial extent of a given region of interest.

Parameters	Estimate	SE	t-value	DF	Uncorrected two-sided p-value	Lower 95% CI	Upper 95% CI
Intercept	0.093	0.040	2.34	100	0.021	0.014	0.17
Age (√year)	0.011	0.0088	1.28	100	0.20	-0.0062	0.029
Number of ASMs	-0.0022	0.0078	-0.28	100	0.78	-0.018	0.013
MRI lesion (yes = 1)	-0.016	0.016	-1.00	100	0.32	-0.048	0.016
Side (left = 1)	0.023	0.015	1.53	100	0.13	-0.0069	0.053
SOZ (parietal = 1)	0.033	0.017	1.97	100	0.051	-1.5×10^{-4}	0.066
Sex (female = 1)	0.018	0.017	1.03	100	0.30	-0.016	0.052

Supplementary Table 24. The results of ancillary mixed model analysis to assess the independent effect of √age on modulation index $(MI)_{\geq 80\ Hz\ \&\ 0.5\text{-}1\ Hz}$ in the posterior cingulate gyrus. ASMs: antiseizure medications. CI: confidence interval. DF: degree of freedom. MI: modulation index. SE: standard error. SOZ: seizure onset zone. **Supplementary Figure 4** shows the spatial extent of a given region of interest.

Parameters	Estimate	SE	t-value	DF	Uncorrected two-sided p-value	Lower 95% CI	Upper 95% CI
Intercept	0.13	0.039	3.24	482	0.0013	0.050	0.21
Age (√year)	0.0071	0.0085	0.84	482	0.40	-0.0096	0.024
Number of ASMs	-0.0084	0.0097	-0.87	482	0.38	-0.027	0.011
MRI lesion (yes = 1)	-0.025	0.017	-1.54	482	0.12	-0.058	0.0070
Side (left = 1)	0.0074	0.016	0.47	482	0.64	-0.023	0.038
SOZ (frontal = 1)	0.031	0.019	1.69	482	0.092	-0.0051	0.068
Sex (female = 1)	0.034	0.015	2.24	482	0.026	0.0042	0.065

Supplementary Table 25. The results of ancillary mixed model analysis to assess the independent effect of √age on modulation index $(MI)_{\geq 80\ Hz\ \&\ 0.5\text{-}1\ Hz}$ in the posterior inferior frontal gyrus. ASMs: antiseizure medications. CI: confidence interval. DF: degree of freedom. MI: modulation index. SE: standard error. SOZ: seizure onset zone. **Supplementary Figure 4** shows the spatial extent of a given region of interest.

Parameters	Estimate	SE	t-value	DF	Uncorrected two-sided p-value	Lower 95% CI	Upper 95% CI
Intercept	0.16	0.037	4.18	365	3.7×10^{-5}	0.083	0.23
Age (√year)	0.0063	0.0082	0.77	365	0.44	-0.0097	0.022
Number of ASMs	-0.018	0.0090	-2.05	365	0.042	-0.036	-7.1×10^{-4}
MRI lesion (yes = 1)	-0.032	0.016	-1.96	365	0.051	-0.064	1.4×10^{-4}
Side (left = 1)	0.011	0.016	0.73	365	0.47	-0.019	0.042
SOZ (frontal = 1)	0.029	0.018	1.58	365	0.11	-0.0070	0.065
Sex (female = 1)	0.023	0.015	1.52	365	0.13	-0.0068	0.054

Supplementary Table 26. The results of ancillary mixed model analysis to assess the independent effect of √age on modulation index (MI)$_{\geq 80\ Hz\ \&\ 0.5\text{-}1\ Hz}$ in the posterior middle frontal gyrus. ASMs: antiseizure medications. CI: confidence interval. DF: degree of freedom. MI: modulation index. SE: standard error. SOZ: seizure onset zone. **Supplementary Figure 4** shows the spatial extent of a given region of interest.

Parameters	Estimate	SE	t-value	DF	Uncorrected two-sided p-value	Lower 95% CI	Upper 95% CI
Intercept	0.15	0.029	5.04	848	5.7×10^{-7}	0.089	0.20
Age (√year)	0.0021	0.0063	0.34	848	0.73	-0.010	0.015
Number of ASMs	-0.010	0.0068	-1.52	848	0.13	-0.024	0.0030
MRI lesion (yes = 1)	-0.014	0.013	-1.14	848	0.25	-0.039	0.010
Side (left = 1)	0.018	0.012	1.52	848	0.13	-0.0052	0.041
SOZ (frontal = 1)	0.013	0.014	0.91	848	0.37	-0.015	0.040
Sex (female = 1)	0.0060	0.011	0.53	848	0.60	-0.016	0.028

Supplementary Table 27. The results of ancillary mixed model analysis to assess the independent effect of √age on modulation index (MI)$_{\geq 80\ Hz\ \&\ 0.5\text{-}1\ Hz}$ in the precentral gyrus. ASMs: antiseizure medications. CI: confidence interval. DF: degree of freedom. MI: modulation index. SE: standard error. SOZ: seizure onset zone. **Supplementary Figure 4** shows the spatial extent of a given region of interest.

Parameters	Estimate	SE	t-value	DF	Uncorrected two-sided p-value	Lower 95% CI	Upper 95% CI
Intercept	0.076	0.052	1.48	149	0.14	-0.026	0.18
Age (√year)	0.025	0.011	2.21	149	0.028	0.0026	0.047
Number of ASMs	0.016	0.014	1.11	149	0.27	-0.012	0.044
MRI lesion (yes = 1)	-0.19	0.024	-0.78	149	0.44	-0.066	0.029
Side (left = 1)	0.0023	0.023	0.10	149	0.92	-0.044	0.048
SOZ (parietal = 1)	-0.011	0.023	-0.50	149	0.62	-0.056	0.034
Sex (female = 1)	0.0090	0.023	0.40	149	0.69	-0.036	0.054

Supplementary Table 28. The results of ancillary mixed model analysis to assess the independent effect of √age on modulation index (MI)$_{\geq 80\ \mathrm{Hz}\ \&\ 0.5\text{-}1\ \mathrm{Hz}}$ in the precuneus. ASMs: antiseizure medications. CI: confidence interval. DF: degree of freedom. MI: modulation index. SE: standard error. SOZ: seizure onset zone. **Supplementary Figure 4** shows the spatial extent of a given region of interest. The observed √age effect did not withstand the false discovery rate correction that was applied for the number of regions of interest.

Parameters	Estimate	SE	t-value	DF	Uncorrected two-sided p-value	Lower 95% CI	Upper 95% CI
Intercept	0.14	0.032	4.3	316	2.2 x 10^{-5}	0.075	0.20
Age (√year)	0.0037	0.0069	0.54	316	0.59	-0.0099	0.017
Number of ASMs	-0.0063	0.0074	-0.85	316	0.39	-0.021	0.0082
MRI lesion (yes = 1)	-0.065	0.014	-4.57	316	7.01 x 10^{-6}	-0.093	-0.037
Side (left = 1)	0.0090	0.014	0.65	316	0.52	-0.018	0.036
SOZ (frontal = 1)	0.030	0.014	2.14	316	0.033	0.0025	0.058
Sex (female = 1)	0.021	0.014	1.48	316	0.14	-0.0067	0.048

Supplementary Table 29. The results of ancillary mixed model analysis to assess the independent effect of √age on modulation index (MI)$_{\geq 80\ \mathrm{Hz}\ \&\ 0.5\text{-}1\ \mathrm{Hz}}$ in the superior frontal gyrus. ASMs: antiseizure medications. CI: confidence interval. DF: degree of freedom. MI: modulation index. SE: standard error. SOZ: seizure onset zone. **Supplementary Figure 4** shows the spatial extent of a given region of interest.

Parameters	Estimate	SE	t-value	DF	Uncorrected two-sided p-value	Lower 95% CI	Upper 95% CI
Intercept	6.7 x 10^{-4}	0.067	0.010	107	0.99	-0.13	0.13
Age (√year)	0.019	0.012	1.54	107	0.13	-0.0054	0.043
Number of ASMs	0.025	0.014	1.71	107	0.090	-0.0039	0.053
MRI lesion (yes = 1)	-0.0057	0.028	-0.20	107	0.84	-0.061	0.050
Side (left = 1)	0.0037	0.026	0.14	107	0.89	-0.047	0.055
SOZ (parietal = 1)	-0.048	0.026	-1.83	107	0.069	-0.099	0.0039
Sex (female = 1)	0.075	0.026	2.87	107	0.0050	0.023	0.13

Supplementary Table 30. The results of ancillary mixed model analysis to assess the independent effect of √age on modulation index (MI)$_{\geq 80\text{ Hz} \,\&\, 0.5\text{-}1\text{ Hz}}$ in the superior parietal lobule. ASMs: antiseizure medications. CI: confidence interval. DF: degree of freedom. MI: modulation index. SE: standard error. SOZ: seizure onset zone. **Supplementary Figure 4** shows the spatial extent of a given region of interest.

Parameters	Estimate	SE	t-value	DF	Uncorrected two-sided p-value	Lower 95% CI	Upper 95% CI
Intercept	0.099	0.021	4.81	714	1.9 x 10^{-6}	0.058	0.14
Age (√year)	0.0052	0.0042	1.25	714	0.21	-0.0030	0.013
Number of ASMs	-0.0018	0.0048	-0.38	714	0.70	-0.011	0.0076
MRI lesion (yes = 1)	-0.0086	0.0087	-0.99	714	0.32	-0.026	0.0085
Side (left = 1)	0.0026	0.0082	0.32	714	0.75	-0.014	0.019
SOZ (temporal = 1)	-0.022	0.0080	-2.78	714	0.0055	-0.038	-0.0065
Sex (female = 1)	0.011	0.0080	1.43	714	0.15	-0.0043	0.027

Supplementary Table 31. The results of ancillary mixed model analysis to assess the independent effect of √age on modulation index (MI)$_{\geq 80\text{ Hz} \,\&\, 0.5\text{-}1\text{ Hz}}$ in the superior temporal gyrus. ASMs: antiseizure medications. CI: confidence interval. DF: degree of freedom. MI: modulation index. SE: standard error. SOZ: seizure onset zone. **Supplementary Figure 4** shows the spatial extent of a given region of interest.

Parameters	Estimate	SE	t-value	DF	Uncorrected two-sided p-value	Lower 95% CI	Upper 95% CI
Intercept	0.12	0.026	4.58	603	5.7×10^{-6}	0.067	0.17
Age (√year)	0.0061	0.0053	1.15	603	0.25	-0.0043	0.017
Number of ASMs	-0.0047	0.0059	-0.80	603	0.43	-0.016	0.0069
MRI lesion (yes = 1)	-0.0083	0.011	-0.76	603	0.45	-0.030	0.013
Side (left = 1)	-0.0018	0.010	-0.18	603	0.86	-0.022	0.018
SOZ (parietal = 1)	-0.020	0.012	-1.66	603	0.097	-0.043	0.0035
Sex (female = 1)	0.023	0.010	2.27	603	0.024	0.0031	0.043

Supplementary Table 32. The results of ancillary mixed model analysis to assess the independent effect of √age on modulation index (MI)$_{\geq 80\ Hz\ \&\ 0.5\text{-}1\ Hz}$ in the supramarginal gyrus. ASMs: antiseizure medications. CI: confidence interval. DF: degree of freedom. MI: modulation index. SE: standard error. SOZ: seizure onset zone. **Supplementary Figure 4** shows the spatial extent of a given region of interest.

Parameters	Estimate	SE	t-value	DF	Uncorrected two-sided p-value	Lower 95% CI	Upper 95% CI
Intercept	0.068	0.027	2.51	142	0.013	0.015	0.12
Age (√year)	6.2×10^{-4}	0.0057	0.11	142	0.91	-0.011	0.012
Number of ASMs	-0.0023	0.0065	-0.35	142	0.73	-0.015	0.011
MRI lesion (yes = 1)	8.7×10^{-5}	0.012	0.0073	142	0.99	-0.023	0.024
Side (left = 1)	0.0063	0.012	0.55	142	0.58	-0.017	0.029
SOZ (temporal = 1)	0.014	0.013	1.08	142	0.28	-0.011	0.039
Sex (female = 1)	0.011	0.011	0.93	142	0.35	-0.012	0.033

Supplementary Table 33. The results of ancillary mixed model analysis to assess the independent effect of √age on modulation index (MI)$_{\geq 80\ Hz\ \&\ 0.5\text{-}1\ Hz}$ in the temporal pole. ASMs: antiseizure medications. CI: confidence interval. DF: degree of freedom. MI: modulation index. SE: standard error. SOZ: seizure onset zone. **Supplementary Figure 4** shows the spatial extent of a given region of interest.

Parameters	Estimate	SE	t-value	DF	Uncorrected two-sided p-value	Lower 95% CI	Upper 95% CI
Intercept	3.27	0.49	6.73	2973	2.1×10^{-11}	2.32	4.22
Age (√year)	-0.53	0.11	-5.01	2973	5.7×10^{-7}	-0.73	-0.32
Number of ASMs	-0.068	0.12	-0.58	2973	0.56	-0.30	0.16
MRI lesion (yes = 1)	-0.23	0.21	-1.09	2973	0.28	-0.64	0.18
Side (left = 1)	-0.11	0.20	-0.56	2973	0.57	-0.50	0.28
SOZ (frontal = 1)	-0.15	0.23	-0.65	2973	0.51	-0.61	0.30
Sex (female = 1)	0.061	0.20	0.31	2973	0.76	-0.32	0.44

Supplementary Table 34. The results of mixed model analysis to assess the independent effect of √age on HFO$_{HIL \geq 80\ Hz}$ in the frontal lobe. ASMs: antiseizure medications. CI: confidence interval. DF: degree of freedom. SE: standard error. SOZ: seizure onset zone.

Parameters	Estimate	SE	t-value	DF	Uncorrected two-sided p-value	Lower 95% CI	Upper 95% CI
Intercept	1.86	0.53	3.54	2388	4.1×10^{-4}	0.83	2.89
Age (√year)	-0.34	0.11	-3.09	2388	0.0020	-0.55	-0.12
Number of ASMs	0.084	0.12	0.69	2388	0.49	-0.15	0.32
MRI lesion (yes = 1)	0.023	0.22	0.10	2388	0.92	-0.41	0.46
Side (left = 1)	-0.042	0.21	-0.20	2388	0.84	-0.45	0.37
SOZ (temporal = 1)	0.32	0.20	1.58	2388	0.12	-0.078	0.72
Sex (female = 1)	-0.038	0.21	-0.18	2388	0.85	-0.44	0.37

Supplementary Table 35. The results of mixed model analysis to assess the independent effect of √age on HFO$_{HIL \geq 80\ Hz}$ in the temporal lobe. ASMs: antiseizure medications. CI: confidence interval. DF: degree of freedom. SE: standard error. SOZ: seizure onset zone.

Parameters	Estimate	SE	t-value	DF	Uncorrected two-sided p-value	Lower 95% CI	Upper 95% CI
Intercept	2.71	0.51	5.26	1994	1.6×10^{-7}	1.70	3.71
Age (√year)	-0.38	0.11	-3.46	1994	5.4×10^{-4}	-0.59	-0.16
Number of ASMs	9.4×10^{-6}	0.12	7.6×10^{-5}	1994	1.00	-0.24	0.24
MRI lesion (yes = 1)	-0.19	0.22	-0.85	1994	0.40	-0.63	0.25
Side (left = 1)	-0.12	0.21	-0.57	1994	0.57	-0.52	0.29
SOZ (parietal = 1)	0.14	0.24	0.61	1994	0.54	-0.32	0.61
Sex (female = 1)	0.20	0.21	0.94	1994	0.35	-0.21	0.60

Supplementary Table 36. The results of mixed model analysis to assess the independent effect of √age on $HFO_{HIL \geq 80\ Hz}$ in the parietal lobe. ASMs: antiseizure medications. CI: confidence interval. DF: degree of freedom. SE: standard error. SOZ: seizure onset zone.

Parameters	Estimate	SE	t-value	DF	Uncorrected two-sided p-value	Lower 95% CI	Upper 95% CI
Intercept	2.31	0.84	2.75	868	0.0062	0.66	3.95
Age (√year)	0.069	0.17	0.40	868	0.69	-0.27	0.41
Number of ASMs	0.073	0.20	0.36	868	0.72	-0.33	0.47
MRI lesion (yes = 1)	0.22	0.36	0.60	868	0.55	-0.49	0.93
Side (left = 1)	-0.66	0.34	-1.93	868	0.054	-1.32	0.011
SOZ (occipital = 1)	1.15	0.44	2.61	868	0.0091	0.29	2.02
Sex (female = 1)	-0.044	0.33	-0.13	868	0.89	-0.70	0.61

Supplementary Table 37. The results of mixed model analysis to assess the independent effect of √age on $HFO_{HIL \geq 80\ Hz}$ in the occipital lobe. ASMs: antiseizure medications. CI: confidence interval. DF: degree of freedom. SE: standard error. SOZ: seizure onset zone.

Parameters	Estimate	SE	t-value	DF	Uncorrected two-sided p-value	Lower 95% CI	Upper 95% CI
Intercept	3.38	0.51	6.64	2890	3.7×10^{-11}	2.38	4.37
Age (√year)	-0.56	0.11	-4.91	2890	9.4×10^{-7}	-0.78	-0.34
Number of ASMs	-0.076	0.12	-0.64	2890	0.52	-0.31	0.16
MRI lesion (yes = 1)	-0.21	0.21	-0.98	2890	0.33	-0.63	0.21
Side (left = 1)	-0.12	0.20	-0.59	2890	0.56	-0.51	0.28
SOZ (frontal = 1)	-0.15	0.23	-0.65	2890	0.52	-0.61	0.31
Sex (female = 1)	0.049	0.20	0.25	2890	0.80	-0.34	0.44

Supplementary Table 38. The results of ancillary mixed model analysis to assess the independent effect of √age on $HFO_{HIL \geq 80\ Hz}$ in the frontal lobe. Here, we present the results of ancillary analysis, excluding three patients of 21 years old and above. ASMs: antiseizure medications. CI: confidence interval. DF: degree of freedom. SE: standard error. SOZ: seizure onset zone.

Parameters	Estimate	SE	t-value	DF	Uncorrected two-sided p-value	Lower 95% CI	Upper 95% CI
Intercept	2.13	0.54	3.95	2309	8.1×10^{-5}	1.07	3.18
Age (√year)	-0.43	0.12	-3.62	2309	3.0×10^{-4}	-0.66	-0.20
Number of ASMs	0.096	0.12	0.79	2309	0.43	-0.14	0.33
MRI lesion (yes = 1)	0.033	0.22	0.15	2309	0.88	-0.40	0.47
Side (left = 1)	-0.11	0.21	-0.54	2309	0.59	-0.52	0.30
SOZ (temporal = 1)	0.39	0.20	1.92	2309	0.055	-0.0088	0.79
Sex (female = 1)	-0.12	0.21	-0.59	2309	0.56	-0.53	0.28

Supplementary Table 39. The results of ancillary mixed model analysis to assess the independent effect of √age on $HFO_{HIL \geq 80\ Hz}$ in the temporal lobe. Here, we present the results of ancillary analysis, excluding three patients of 21 years old and above. ASMs: antiseizure medications. CI: confidence interval. DF: degree of freedom. SE: standard error. SOZ: seizure onset zone.

Parameters	Estimate	SE	t-value	DF	Uncorrected two-sided p-value	Lower 95% CI	Upper 95% CI
Intercept	2.93	0.53	5.49	1944	4.5×10^{-8}	1.89	3.98
Age (√year)	-0.45	0.12	-3.79	1944	1.5×10^{-4}	-0.68	-0.22
Number of ASMs	-2.5×10^{-4}	0.12	-0.0020	1944	1.00	-0.24	0.24
MRI lesion (yes = 1)	-0.19	0.23	-0.83	1944	0.41	-0.63	0.26
Side (left = 1)	-0.15	0.21	-0.74	1944	0.46	-0.56	0.26
SOZ (parietal = 1)	0.17	0.24	0.71	1944	0.48	-0.30	0.63
Sex (female = 1)	0.15	0.21	0.70	1944	0.48	-0.27	0.56

Supplementary Table 40. The results of ancillary mixed model analysis to assess the independent effect of √age on $HFO_{HIL \geq 80\ Hz}$ in the parietal lobe. Here, we present the results of ancillary analysis, excluding three patients of 21 years old and above. ASMs: antiseizure medications. CI: confidence interval. DF: degree of freedom. SE: standard error. SOZ: seizure onset zone.

Parameters	Estimate	SE	t-value	DF	Uncorrected two-sided p-value	Lower 95% CI	Upper 95% CI
Intercept	2.14	0.89	2.40	826	0.017	0.39	3.88
Age (√year)	0.11	0.19	0.60	826	0.55	-0.26	0.49
Number of ASMs	0.059	0.21	0.28	826	0.78	-0.35	0.47
MRI lesion (yes = 1)	0.23	0.37	0.61	826	0.54	-0.50	0.95
Side (left = 1)	-0.60	0.35	-1.72	826	0.086	-1.29	0.086
SOZ (occipital = 1)	1.22	0.47	2.60	826	0.0094	0.30	2.14
Sex (female = 1)	0.0090	0.34	0.026	826	0.98	-0.67	0.68

Supplementary Table 41. The results of ancillary mixed model analysis to assess the independent effect of √age on $HFO_{HIL \geq 80\ Hz}$ in the occipital lobe. Here, we present the results of ancillary analysis, excluding three patients of 21 years old and above. ASMs: antiseizure medications. CI: confidence interval. DF: degree of freedom. SE: standard error. SOZ: seizure onset zone.

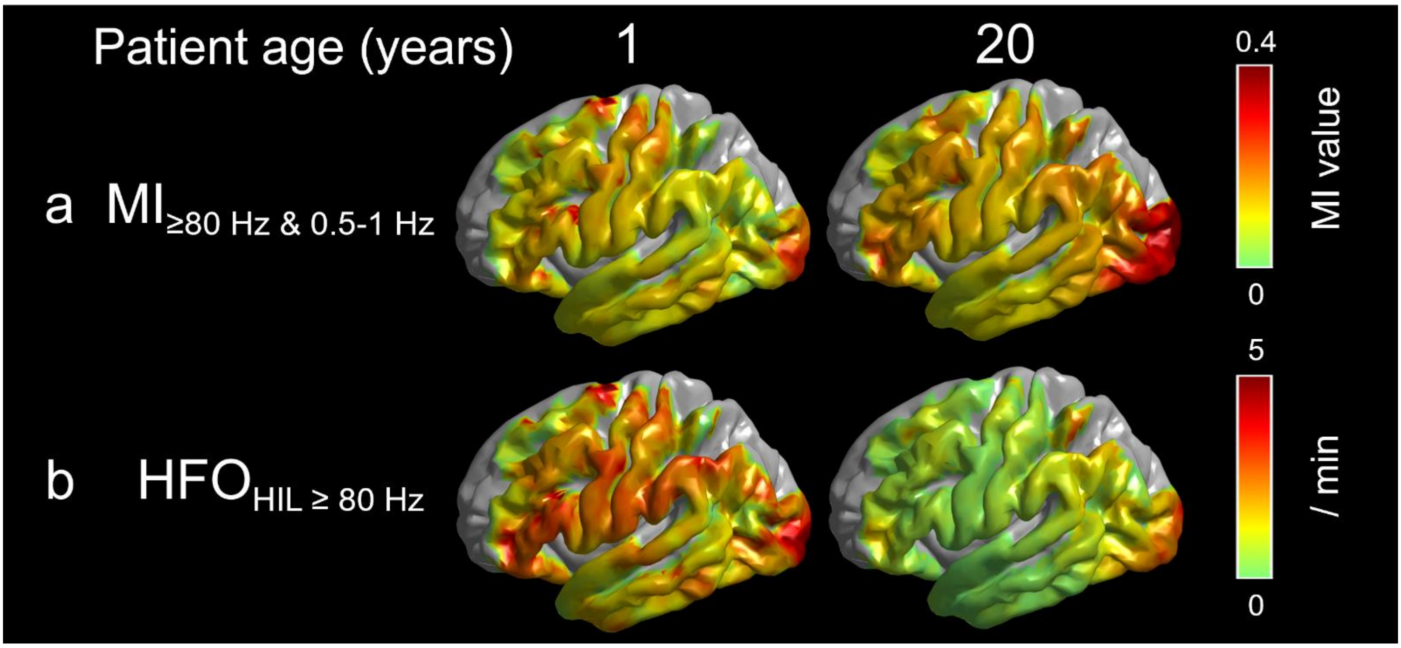

Supplementary Figure 1. Summary observations.
a Our normative atlas reveals a marked enhancement of modulation index (MI) in the nonepileptic occipital lobe compared to the other lobes across childhood, and occipital MI shows an age-related increase particularly during early childhood. $MI_{\geq 80\text{ Hz and }0.5\text{-}1\text{ Hz}}$ denotes the strength of coupling between the amplitude of high-frequency oscillation (HFO) at ≥80 Hz and the phase of slow-wave at 0.5-1 Hz. **b** The HFO rate in the nonepileptic occipital area exhibits no significant correlation with age, whereas each of the other three brain lobes demonstrates an inverse relationship with age. The brain images in this figure were created using FreeSurfer (https://surfer.nmr.mgh.harvard.edu/fswiki/CorticalParcellation). HFO_{HIL}: HFO rate defined by the Hilbert method.

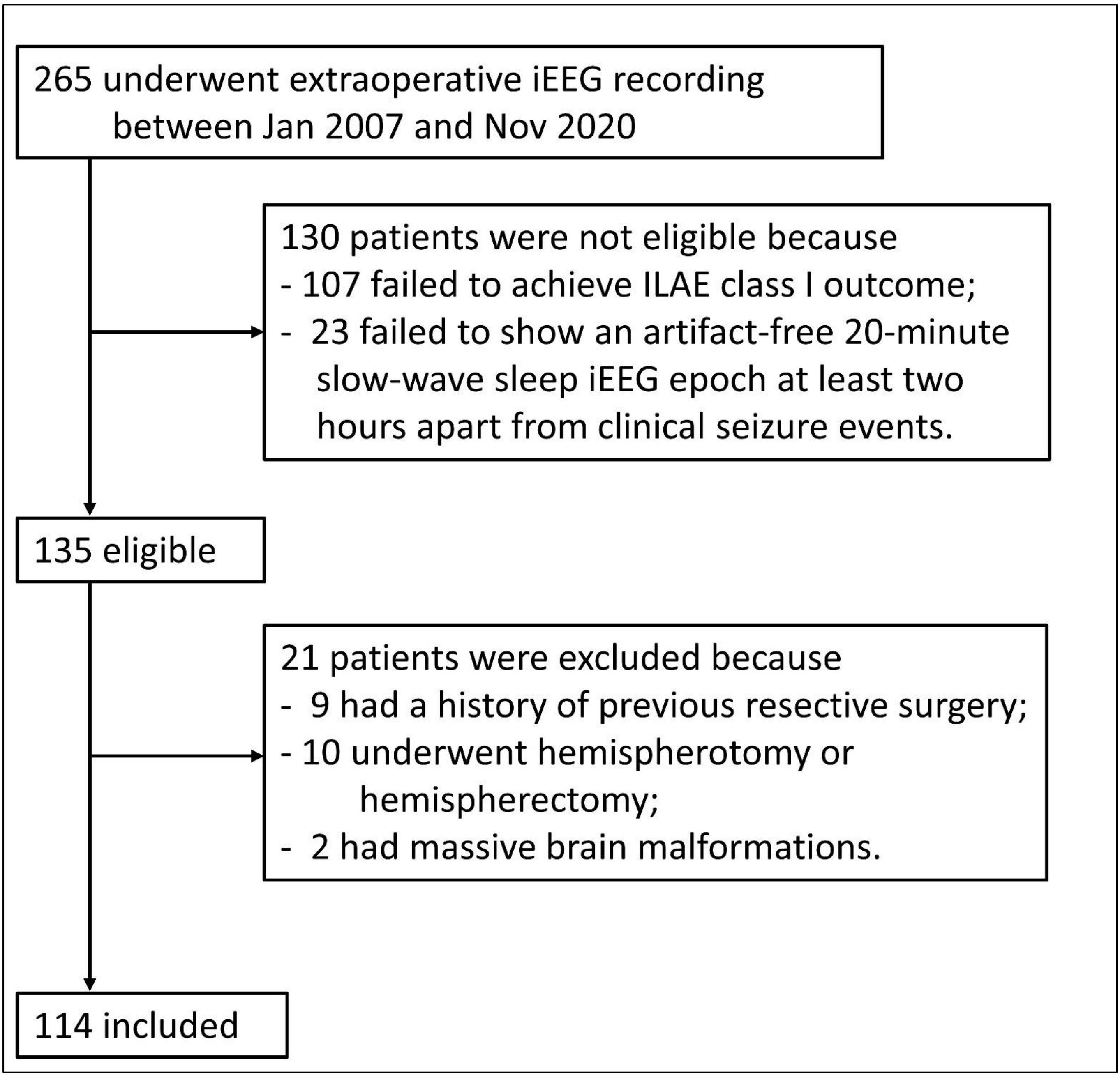

Supplementary Figure 2. Flowcharts showing the study patients satisfying the eligibility criteria. Out of the 265 patients with a diagnosis of drug-resistant focal epilepsy, 130 patients failed to satisfy the inclusion criteria. Specifically, 107 patients failed to achieve the ILAE class 1 outcome following surgery, whereas 23 patients failed to show an artifact-free 20-minute slow-wave sleep intracranial EEG epoch at least two hours apart from clinical seizure events. Out of the 135 patients satisfying the inclusion criteria, 21 patients were excluded because nine had a history of previous resective surgery, 10 underwent either hemispherotomy or hemispherectomy, and the remaining two had massive brain malformations, making it difficult to identify the central, lateral, or calcarine sulci. As a result, a total of 114 patients satisfying the eligibility criteria were studied. iEEG: intracranial electroencephalography. ILAE: International League Against Epilepsy.

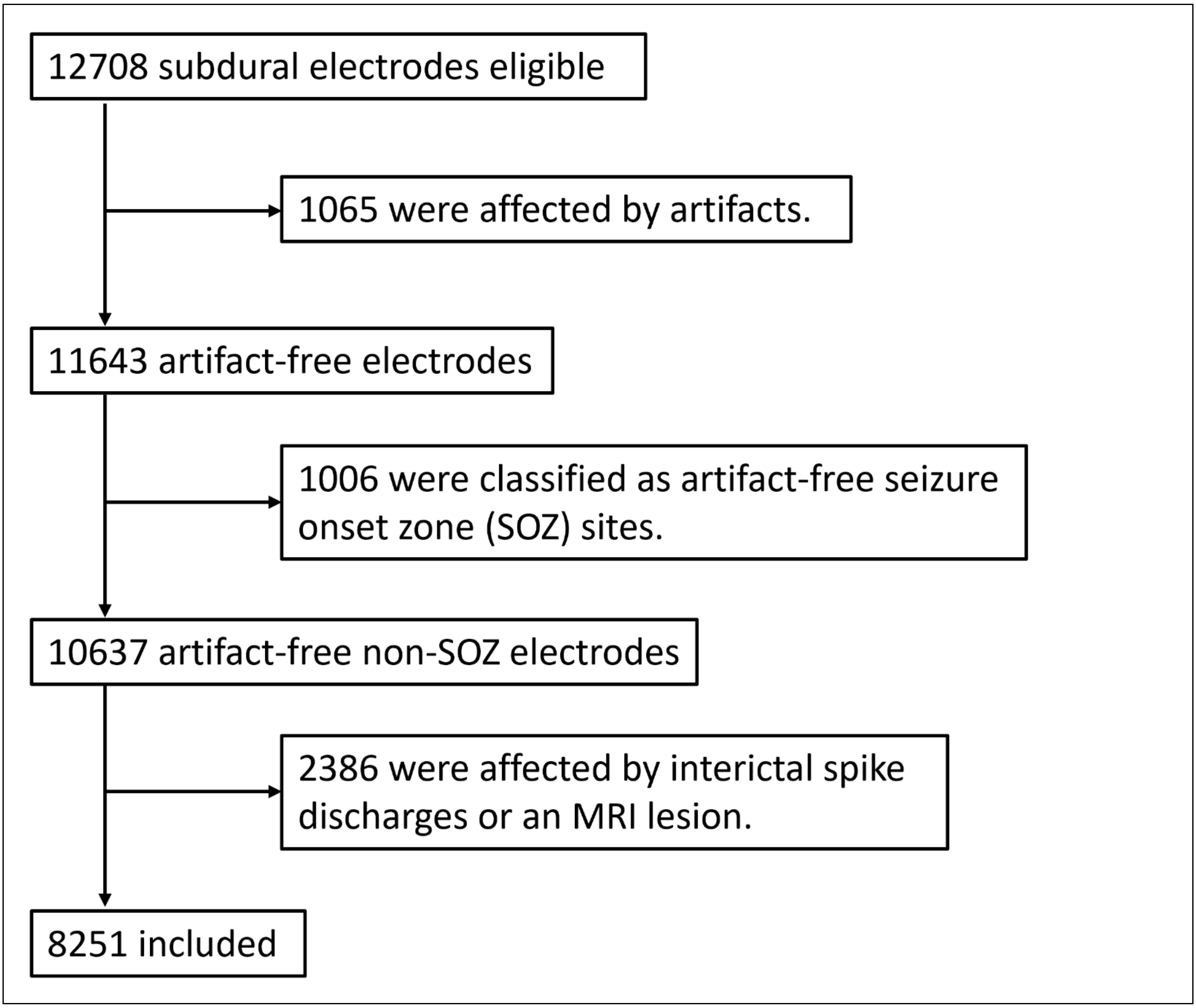

Supplementary Figure 3. Flowcharts showing the number of subdural electrodes satisfying the eligibility criteria of artifact-free nonepileptic electrode sites.

The flowchart illustrates the process of identifying the number of subdural electrodes that satisfy the eligibility criteria of being artifact-free nonepileptic electrode sites. Initially, out of the 12708 subdural electrode sites, 1065 sites were affected by artifacts. From the remaining 11643 sites, 1006 were classified as artifact-free seizure onset zone (SOZ) sites. Of the 10637 remaining artifact-free non-SOZ sites, 2386 were affected by interictal spike discharges or an MRI lesion. Thus, a total of 8251 sites were considered to be artifact-free nonepileptic electrode sites.

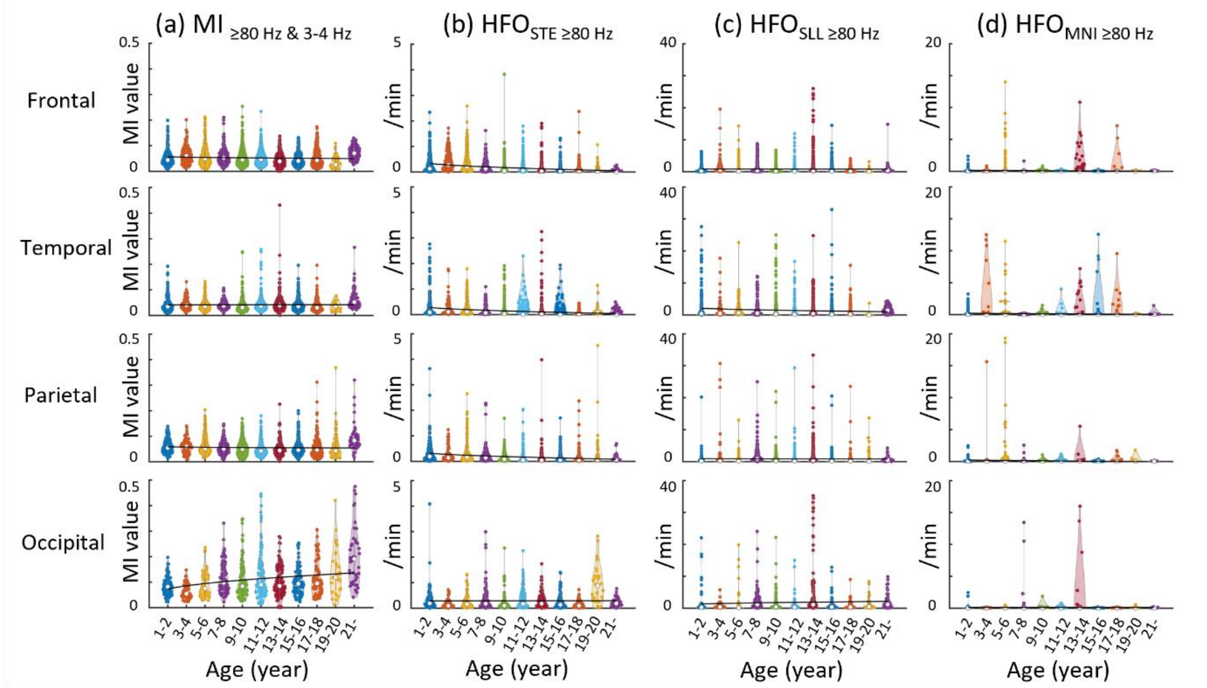

Supplementary Figure 4. The developmental changes of cortical MI and HFO at given lobes. **a** $MI_{\geq 80\ Hz\ \&\ 3\text{-}4\ Hz}$: the strength of coupling between the amplitude of $HFO_{\geq 80\ Hz}$ and the phase of slow-$wave_{3\text{-}4\ Hz}$, as rated by modulation index. **b** $HFO_{STE\ \geq 80\ Hz}$ occurrence rate (/min). **c** $HFO_{SLL\ \geq 80\ Hz}$ occurrence rate. **d** $HFO_{MNI\ \geq 80\ Hz}$ occurrence rate. In each violin plot, a regression line is provided based on a model incorporating the square root of age (√age) as an independent variable. The white circle within each violin plot represents the median. MI: modulation index. HFO_{STE}: high-frequency oscillation (HFO) defined by Staba et al.[1]. HFO_{SLL}: HFO defined by Gardner et al.[2]. HFO_{HIL}: HFO defined by Crépon et al.[3]. HFO_{MNI}: HFO defined by Zelmann et al.[4].

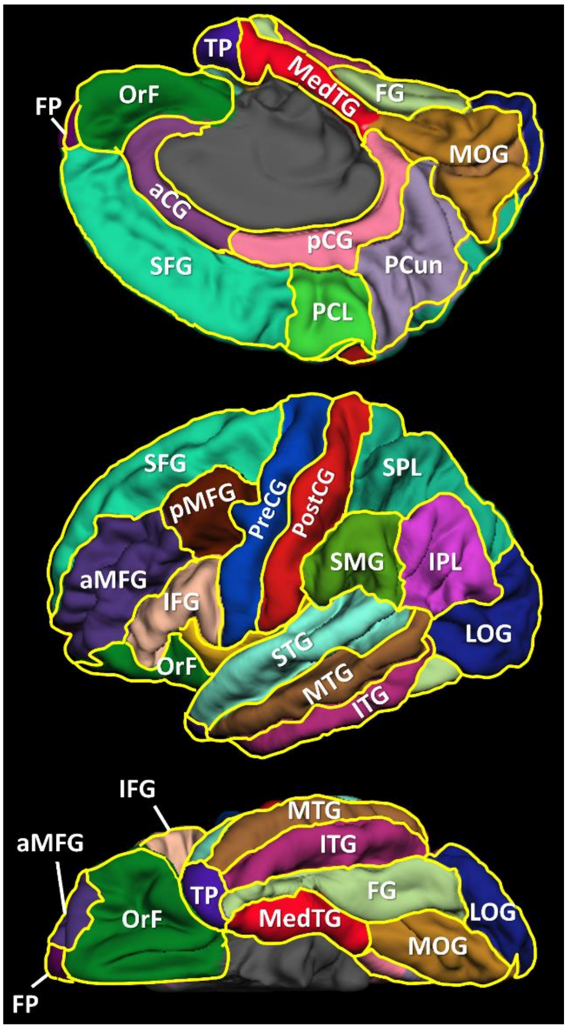

Supplementary Figure 5. Regions of interest (ROIs). ROI locations on the left hemisphere are presented. aCG: anterior cingulate gyrus. aMFG: anterior middle-frontal gyrus. FG: fusiform gyrus. IFG: inferior-frontal gyrus (summation of pars opercularis and pars triangularis). IPL: inferior parietal lobule. ITG: inferior-temporal gyrus. LOG: lateral occipital gyrus. MedTG: medial-temporal gyrus (summation of entorhinal and parahippocampal gyri). MOG: medial occipital gyrus (summation of cuneus and lingual gyri). MTG: middle-temporal gyrus. OrF: orbitofrontal gyrus (summation of pars orbitalis and medial and lateral orbitofrontal gyri). pCG: posterior cingulate gyrus. PCL: paracentral lobule. PCun: Precuneus gyrus. pMFG: posterior middle-frontal gyrus. PoCG: postcentral gyrus. PreCG: precentral gyrus. SFG: superior-frontal gyrus. SMG: supramarginal gyrus. SPL: superior parietal lobule. STG: superior-temporal gyrus. TP: temporal pole. A total of 22 regions mentioned above in both hemispheres were included in the ROI-based analysis. The following electrode site was not included in the group-level ROI analysis because the number of electrode sites was below five (see below). FP: frontal pole. The aforementioned ROIs[5,6] were used in the ancillary mixed model analysis to assess the independent effect of $\sqrt{\text{age}}$ on modulation index $(\text{MI})_{\geq 80\ \text{Hz}\ \&\ 0.5\text{-}1\ \text{Hz}}$ in a given region (**Supplementary Tables 20-33**). The brain images in this figure were created using FreeSurfer (https://surfer.nmr.mgh.harvard.edu/fswiki/CorticalParcellation).

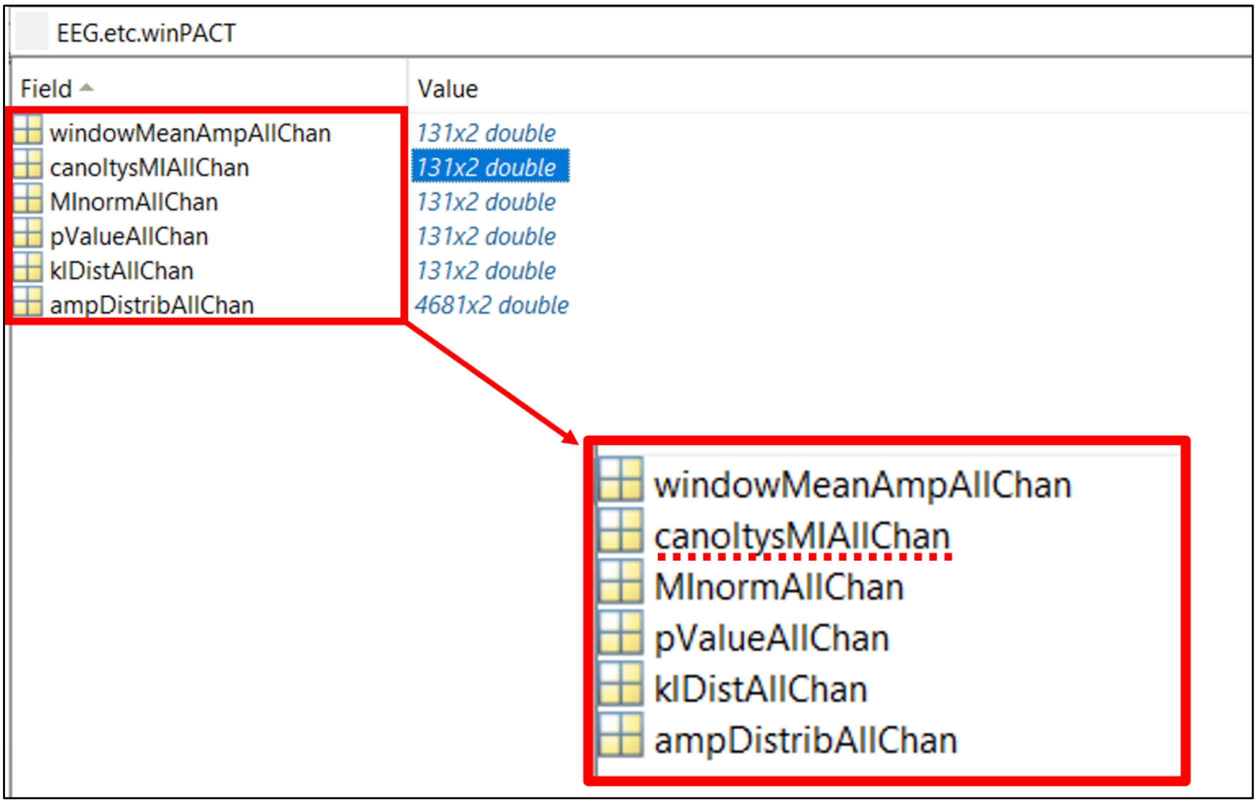

Supplementary Figure 6. Computation of modulation index. To compute the modulation index, we used the EEG.etc.winPACT.canoltysMIAllChan command within the winPACT toolbox (https://github.com/sccn/winPACT).

Supplementary References

1. Staba, R. J., Wilson, C. L., Bragin, A., Fried, I. & Engel, J. Jr. Quantitative analysis of high-frequency oscillations (80-500 Hz) recorded in human epileptic hippocampus and entorhinal cortex. *J Neurophysiol*. **88**, 1743-1752 (2002).

2. Gardner, A. B., Worrell, G. A., Marsh, E., Dlugos, D. & Litt, B. Human and automated detection of high-frequency oscillations in clinical intracranial EEG recordings. *Clin Neurophysiol*. **118**, 1134-1143 (2007).

3. Crépon, B. et al. Mapping interictal oscillations greater than 200 Hz recorded with intracranial macroelectrodes in human epilepsy. *Brain*. **133**, 33-45 (2010).

4. Zelmann, R. et al. Automatic detector of high frequency oscillations for human recordings with macroelectrodes. *Annu Int Conf IEEE Eng Med Biol Soc*. **2010**, 2329-2333 (2010).

5. Desikan, R. S. et al. An automated labeling system for subdividing the human cerebral cortex on MRI scans into gyral based regions of interest. *Neuroimage*. **31**, 968-980 (2006).

6. Kitazawa, Y. et al. Intra- and inter-hemispheric network dynamics supporting object recognition and speech production. *Neuroimage*. **270**, 119954 (2023).
