# Supplementary material for: Developmental atlas of phase-amplitude coupling between physiologic high-frequency oscillations and slow waves
Source: Nat Commun. 2023 Oct 13;14:6435. doi: 10.1038/s41467-023-42091-y (PMC10575956; doi:10.1038/s41467-023-42091-y)
Supplement: Supplementary file 3 — Description of Additional Supplementary Files [file 41467_2023_42091_MOESM3_ESM.pdf]

Description of Additional Supplementary Files

File name: Supplementary Movie 1

Description: The developmental atlases of cortical $MI_{\geq 80\ Hz\ and\ 0.5\text{-}1\ Hz}$. This video offers a thorough overview of normative MI development at specific mesh points, estimated by univariate regression models incorporating age, √age, or log_{10} age as independent variables. Violin plots depicting the developmental changes of MI at given lobes are also presented, along with their corresponding regression slope, uncorrected p-value, regression t-value, and degree of freedom (DF). The brain images in this movie were created using FreeSurfer (https://surfer.nmr.mgh.harvard.edu/fswiki/CorticalParcellation).

Timeline

0:00-0:40. Based on the regression model as a function of √age.

0:40-1:20. Based on the regression model as a function of log_{10} age.

1:20-2:00. Based on the regression model as a function of age.

File name: Supplementary Movie 2

Description: The developmental atlases of cortical $MI_{\geq 80\ Hz\ and\ 3\text{-}4\ Hz}$. This video offers a thorough overview of normative MI development at specific mesh points, estimated by univariate regression models incorporating age, √age, or log_{10} age as independent variables. Violin plots depicting the developmental changes of MI at given lobes are also presented, along with their corresponding regression slope, uncorrected p-value, regression t-value, and degree of freedom (DF). The brain images in this movie were created using FreeSurfer (https://surfer.nmr.mgh.harvard.edu/fswiki/CorticalParcellation).

Timeline

0:00-0:40. Based on the regression model as a function of √age.

0:40-1:20. Based on the regression model as a function of log_{10} age.

1:20-2:00. Based on the regression model as a function of age.

File name: Supplementary Movie 3

Description: The developmental atlases of cortical $HFO_{HIL\ \geq 80\ Hz}$. This video offers a thorough overview of normative HFO development at specific mesh points, estimated by univariate regression models incorporating age, √age, or log_{10} age as independent variables. Violin plots depicting the developmental changes of MI at given lobes are also presented, along with their corresponding regression slope, uncorrected p-value, regression t-value, and degree of freedom (DF). The brain images in this movie were created using FreeSurfer

(https://surfer.nmr.mgh.harvard.edu/fswiki/CorticalParcellation).
Timeline
0:00-0:40. Based on the regression model as a function of √age.
0:40-1:20. Based on the regression model as a function of $\log_{10}$ age.
1:20-2:00. Based on the regression model as a function of age.

File name: Supplementary Movie 4

Description: Dynamic tractography of $MI_{\geq 80\ Hz\ and\ 0.5\text{-}1\ Hz}$. The video presents the intensity of developmental co-growth of $MI_{\geq 80\ Hz\ \&\ 0.5\text{-}1\ Hz}$, as predicted by univariate regression model incorporating √age and $\log_{10}$ age as an independent variable. The brain images in this movie were created using FreeSurfer (https://surfer.nmr.mgh.harvard.edu/fswiki/CorticalParcellation).
Timeline
0:00-0:25. Based on the regression model as a function of √age.
0:25-0:50. Based on the regression model as a function of $\log_{10}$ age.

File name: Supplementary Movie 5

Description: Dynamic tractography of $HFO_{HIL\geq 80\ Hz}$. The video presents the intensity of developmental co-diminution of $HFO_{HIL\ \geq 80\ Hz}$, as predicted by univariate regression model incorporating √age and $\log_{10}$ age as an independent variable. The brain images in this movie were created using FreeSurfer (https://surfer.nmr.mgh.harvard.edu/fswiki/CorticalParcellation).
Timeline
0:00-0:25. Based on the regression model as a function of √age.
0:25-0:50. Based on the regression model as a function of $\log_{10}$ age.

File name: Supplementary Movie 6

Description: The normative ranges of cortical $MI_{\geq 80\ Hz\ and\ 0.5\text{-}1\ Hz}$ and $MI_{\geq 80\ Hz\ and\ 3\text{-}4\ Hz}$. On the left side, the video presents the mean of normative MI at given mesh points, as estimated by the univariate regression model incorporating √age. On the right side, it presents the mean plus two standard deviations, likewise estimated by the univariate regression model. For children who are 'n' years old, we computed the standard deviation across children between 'n' and 'n+3.9' years old. The brain images in this movie were created using FreeSurfer (https://surfer.nmr.mgh.harvard.edu/fswiki/CorticalParcellation).
Timeline
00:00-00:25 $MI_{\geq 80\ Hz\ and\ 0.5\text{-}1\ Hz}$.
00:25-00:50 $MI_{\geq 80\ Hz\ and\ 3\text{-}4\ Hz}$.

File name: Supplementary Movie 7

Description: The normative ranges of cortical $MI_{\geq 150\ Hz\ and\ 0.5\text{-}1\ Hz}$ and $MI_{\geq 150\ Hz\ and\ 3\text{-}4\ Hz}$. On the left side, the video presents the mean of normative MI at given mesh points, as estimated by the univariate regression model incorporating √age. On the right side, it presents the mean plus two standard deviations, likewise estimated by the univariate regression model. For children who are 'n' years old, we computed the standard deviation across children between 'n' and 'n+3.9' years old. The brain images in this movie were created using FreeSurfer (https://surfer.nmr.mgh.harvard.edu/fswiki/CorticalParcellation).

Timeline

00:00-00:25 $MI_{\geq 150\ Hz\ and\ 0.5\text{-}1\ Hz}$.

00:25-00:50 $MI_{\geq 150\ Hz\ and\ 3\text{-}4\ Hz}$.

File name: Supplementary Movie 8

Description: The normative range of cortical $HFO_{HIL\ \geq 80\ Hz}$. On the left side, the video presents the mean of normative $HFO_{HIL\ \geq 80\ Hz}$ at given mesh points, as estimated by the univariate regression model incorporating √age. On the right side, it presents the mean plus two standard deviations, likewise estimated by the univariate regression model. For children who are 'n' years old, we computed the standard deviation across children between 'n' and 'n+3.9' years old. The brain images in this movie were created using FreeSurfer (https://surfer.nmr.mgh.harvard.edu/fswiki/CorticalParcellation).
