# Supplementary material for: Developmental atlas of phase-amplitude coupling between physiologic high-frequency oscillations and slow waves
Source: Nat Commun. 2023 Oct 13;14:6435. doi: 10.1038/s41467-023-42091-y (PMC10575956; doi:10.1038/s41467-023-42091-y)
Supplement: Supplementary file 12 — Reporting Summary [file 41467_2023_42091_MOESM12_ESM.pdf]

Corresponding author(s): Eishi Asano, MD, PhD

Last updated by author(s): Aug 21, 2023

Reporting Summary

Nature Portfolio wishes to improve the reproducibility of the work that we publish. This form provides structure for consistency and transparency in reporting. For further information on Nature Portfolio policies, see our Editorial Policies and the Editorial Policy Checklist.

Statistics

For all statistical analyses, confirm that the following items are present in the figure legend, table legend, main text, or Methods section.

n/a	Confirmed	
☐	☒	The exact sample size (*n*) for each experimental group/condition, given as a discrete number and unit of measurement
☐	☒	A statement on whether measurements were taken from distinct samples or whether the same sample was measured repeatedly
☐	☒	The statistical test(s) used AND whether they are one- or two-sided<br>*Only common tests should be described solely by name; describe more complex techniques in the Methods section.*
☐	☒	A description of all covariates tested
☐	☒	A description of any assumptions or corrections, such as tests of normality and adjustment for multiple comparisons
☐	☒	A full description of the statistical parameters including central tendency (e.g. means) or other basic estimates (e.g. regression coefficient) AND variation (e.g. standard deviation) or associated estimates of uncertainty (e.g. confidence intervals)
☐	☒	For null hypothesis testing, the test statistic (e.g. *F*, *t*, *r*) with confidence intervals, effect sizes, degrees of freedom and *P* value noted<br>*Give P values as exact values whenever suitable.*
☒	☐	For Bayesian analysis, information on the choice of priors and Markov chain Monte Carlo settings
☐	☒	For hierarchical and complex designs, identification of the appropriate level for tests and full reporting of outcomes
☐	☒	Estimates of effect sizes (e.g. Cohen's *d*, Pearson's *r*), indicating how they were calculated

Our web collection on statistics for biologists contains articles on many of the points above.

Software and code

Policy information about availability of computer code

Data collection	[1] Intracranial EEG (iEEG) data: Neurofax 1100A Digital System (Nihon Kohden America Inc., Foothill Ranch, CA, USA). [2] T1-weighted MRI imaging and diffusion-weighted imaging: GE Signa 3T scanner (GE Healthcare, Milwaukee, WI, USA).
Data analysis	[1] EEGLAB Toolbox winPACT (https://sccn.ucsd.edu/wiki/WinPACT) for computing the modulation index. [2] RIPPLELAB software (https://github.com/BSP-Uniandes/RIPPLELAB/) for computing the rate of high-frequency oscillations. [3] FreeSurfer version 6.0.0 (https://surfer.nmr.mgh.harvard.edu/) for processing and analyzing MRI. [4] DSI studio (http://dsi-studio.labsolver.org/) for dynamic tractography analysis. [5] Statistics implemented in the MATLAB version R2020a.

For manuscripts utilizing custom algorithms or software that are central to the research but not yet described in published literature, software must be made available to editors and reviewers. We strongly encourage code deposition in a community repository (e.g. GitHub). See the Nature Portfolio guidelines for submitting code & software for further information.

Data

Policy information about availability of data

All manuscripts must include a data availability statement. This statement should provide the following information, where applicable:

- Accession codes, unique identifiers, or web links for publicly available datasets
- A description of any restrictions on data availability
- For clinical datasets or third party data, please ensure that the statement adheres to our policy

We stated "The iEEG data are available at https://openneuro.org/datasets/ds004551/versions/1.0.6 (doi:10.18112/openneuro.ds004551.v1.0.6)" in Data availability section. We also stated "The analysis codes are available at https://github.com/kaz1126/MI_HFO (doi.org/10.5281/zenodo.8267570)" in Code availability section.

Research involving human participants, their data, or biological material

Policy information about studies with human participants or human data. See also policy information about sex, gender (identity/presentation), and sexual orientation and race, ethnicity and racism.

Reporting on sex and gender	We examined individuals diagnosed with focal epilepsy, encompassing both female and male patients (i.e., female: 54 patients; male: 60 patients). Table 1 denotes the total number of females and males solely based on their biological attribute. We obtained sex information via patient's chart, whereas our study did not involve any gender-based analysis. We employed the mixed model analysis to consider the potential effect of sex on intracranial EEG measures.
Reporting on race, ethnicity, or other socially relevant groupings	We did not use race, ethnicity, or other socially relevant groupings in any of the analyses.
Population characteristics	We studied a consecutive series of 114 patients with focal epilepsy (ages 1.0 to 41.5 years) who met the following eligibility criteria (Table 1; Table S1). The inclusion criteria consisted of [a] simultaneous video-iEEG recording between January 2007 and November 2020, as part of presurgical assessment at Children's Hospital of Michigan or Harper University Hospital, Detroit Medical Center, [b] iEEG sampling rate of 1,000 Hz, [c] iEEG contained an artifact-free 20-minute slow-wave sleep epoch at least two hours apart from clinical seizure events, and [d] International League Against Epilepsy (ILAE) class 1 outcome in the last follow-up after focal resection. The exclusion criteria included [a] a history of previous resective epilepsy surgery, [b] undergoing hemispherotomy or hemispherectomy, [c] lacking artifact-free, nonepileptic electrode sites (defined as those outside the SOZ, interictal spike zone, and MRI-visible lesions), and [d] the presence of massive brain malformations (such as megalencephaly and perisylvian polymicrogyria) that make it difficult to reliably identify the central, lateral, or calcarine sulci.
Recruitment	All patients were diagnosed with drug-resistant focal epilepsy and recruited from the Neurology/Neurosurgery Clinics at the Children's Hospital of Michigan in Detroit, USA. All procedures, including intracranial EEG recording, were undertaken as part of standard clinical management. Written informed consent was obtained from each patient's parents or legal guardians prior to the surgical procedure. Assent was also obtained from patients aged over 13 years. It's essential to note that it is unethical to implant subdural electrodes without a clinical indication. To ensure the validity of our normative atlas, we excluded electrode sites impacted by the seizure onset zone, interictal spike zone, or MRI-visible lesions from the normative atlas. There were variations among participants regarding the number of anti-seizure medications, localizations of seizure onset zones, and MRI-visible structural lesions. These variables are potential sources of bias. To counteract this bias, we utilized a mixed model analysis to control for potential confounding factors affecting intracranial EEG measures. These factors included anti-seizure medications, presence of MRI-visible lesions, and seizure onset zone locations. As a result, we characterized developmental changes in intracranial EEG, factoring out these potential confounders.
Ethics oversight	This study has been approved by the Wayne State University Institutional Review Board (048404MP2E).

Note that full information on the approval of the study protocol must also be provided in the manuscript.

Field-specific reporting

Please select the one below that is the best fit for your research. If you are not sure, read the appropriate sections before making your selection.

☒ Life sciences ☐ Behavioural & social sciences ☐ Ecological, evolutionary & environmental sciences

For a reference copy of the document with all sections, see nature.com/documents/nr-reporting-summary-flat.pdf

Life sciences study design

All studies must disclose on these points even when the disclosure is negative.

Sample size	iEEG signals recorded from 8,251 nonepileptic cortical sites (114 patients; aged: 1.0-41.5 years). We studied a consecutive series of 114 patients with focal epilepsy (ages 1.0 to 41.5 years) who met the following eligibility criteria (Table 1; Table S1). The inclusion criteria consisted of [a] simultaneous video-iEEG recording between January 2007 and November 2020, as part of presurgical assessment at Children's Hospital of Michigan or Harper University Hospital, Detroit Medical Center, [b] iEEG sampling rate of 1,000 Hz, [c] iEEG contained an artifact-free 20-

minute slow-wave sleep epoch at least two hours apart from clinical seizure events, and [d] International League Against Epilepsy (ILAE) class 1 outcome in the last follow-up after focal resection.

Data exclusions

The exclusion criteria included [a] a history of previous resective epilepsy surgery, [b] undergoing hemispherotomy or hemispherectomy, [c] lacking artifact-free, nonepileptic electrode sites (defined as those outside the SOZ, interictal spike zone, and MRI-visible lesions), and [d] the presence of massive brain malformations (such as megalencephaly and perisylvian polymicrogyria) that make it difficult to reliably identify the central, lateral, or calcarine sulci.

Replication

We replicated the experiments at least twice. We have confirmed that all attempts at replication were successful.

Randomization

In this observational study, we did not employ randomization. All data were derived from the standard treatment of drug-resistant focal epilepsy, with no deviation from clinical protocols.

Blinding

In this observational study, we did not employ blinding. All data were derived from the standard treatment of drug-resistant focal epilepsy, with no deviation from clinical protocols.

Reporting for specific materials, systems and methods

We require information from authors about some types of materials, experimental systems and methods used in many studies. Here, indicate whether each material, system or method listed is relevant to your study. If you are not sure if a list item applies to your research, read the appropriate section before selecting a response.

Materials & experimental systems

n/a	Involved in the study
☒	☐ Antibodies
☒	☐ Eukaryotic cell lines
☒	☐ Palaeontology and archaeology
☒	☐ Animals and other organisms
☐	☒ Clinical data
☒	☐ Dual use research of concern
☒	☐ Plants

Methods

n/a	Involved in the study
☒	☐ ChIP-seq
☒	☐ Flow cytometry
☐	☒ MRI-based neuroimaging

Clinical data

Policy information about clinical studies

All manuscripts should comply with the ICMJE guidelines for publication of clinical research and a completed CONSORT checklist must be included with all submissions.

Clinical trial registration

This observational study was not registered to ClinicalTrials.gov. This study aims to provide the normative developmental atlas of intracranial EEG measures.

Study protocol

We studied a consecutive series of 114 patients with focal epilepsy (ages 1.0 to 41.5 years) who met the following eligibility criteria (Table 1; Table S1). The inclusion criteria consisted of [a] simultaneous video-iEEG recording between January 2007 and November 2020, as part of presurgical assessment at Children's Hospital of Michigan or Harper University Hospital, Detroit Medical Center, [b] iEEG sampling rate of 1,000 Hz, [c] iEEG contained an artifact-free 20-minute slow-wave sleep epoch at least two hours apart from clinical seizure events, and [d] International League Against Epilepsy (ILAE) class 1 outcome in the last follow-up after focal resection. The exclusion criteria included [a] a history of previous resective epilepsy surgery, [b] undergoing hemispherotomy or hemispherectomy, [c] lacking artifact-free, nonepileptic electrode sites (defined as those outside the SOZ, interictal spike zone, and MRI-visible lesions), and [d] the presence of massive brain malformations (such as megalencephaly and perisylvian polymicrogyria) that make it difficult to reliably identify the central, lateral, or calcarine sulci. The Institutional Review Board of Wayne State University approved the current study, and we obtained written informed consent from the patients' legal guardians and written assent from pediatric patients aged 13 years or older. We acquired extraoperative video-iEEG data using the same protocols as reported in our previous studies (Nagasawa et al., 2012; Kuroda et al., 2021; Sakakura et al., 2022). A licensed neurosurgeon surgically implanted platinum subdural disk electrodes (3 mm diameter and 10 mm center-to-center distance) on the hemisphere suspected to contain the epileptogenic zone, followed by the placement of surface electromyographic electrodes on the deltoid muscles and electrooculographic electrodes (2.5 cm below and 2.5 cm lateral to the outer canthi) to assess body movements during iEEG recording (Nariai et al., 2011; Nagasawa et al., 2012). Intracranial EEG recording aimed to determine the boundaries of the presumed epileptogenic zone and functionally important cortices, and the spatial extent and duration of iEEG sampling were solely determined by clinical needs specific to each patient. Following implantation, iEEG data was continuously recorded at the bedside with a sampling rate of 1,000 Hz, for 2-7 days. We obtained preoperative 3-tesla MRI data, including T1-weighted spoiled gradient-echo volumetric and fluid-attenuated inversion recovery images (Nakai et al., 2017; Sakakura et al., 2022). We displayed electrode sites on the pial surface of the brain using preoperative MRI and a post-implant CT image (Stolk et al., 2018; Sakakura et al., 2022). Two board-certified neurosurgeons (K.S. and N.K.) visually assessed intraoperative photographs to confirm the spatial accuracy of electrode locations co-registered to the MRI surface image (Pieters et al., 2013). In our previous studies (Asano et al., 2009; Sonoda et al., 2022), we described the guiding principle for determining the boundary of cortical resection. Our aim was to remove the SOZ and any adjacent MRI lesions, while preserving functionally-important cortex; this procedure is intended to maximize seizure control and minimize development of cognitive and/or sensorimotor deficits. Importantly, none of the data from this study was available to inform clinical decision making. Consistent with the study design, all 114 patients achieved ILAE Class-1 outcome at the last follow-up, which occurred at least one year after surgery.

Data collection

All patients were recruited at the Neurology/Neurosurgery Clinics at Children's Hospital of Michigan in Detroit, USA. Prior to the surgical procedure, written informed consent was obtained from each patient's parents or legal guardians, and assent was obtained from patients older than 13 years.

Outcomes

The current study aimed to build the normative developmental atlas of intracranial EEG measures. Consistent with the study design, all 114 patients achieved ILAE Class-1 seizure outcome at the last follow-up, which occurred at least one year after surgery.

Magnetic resonance imaging

Experimental design

Design type

Resting state. Structural MRI was acquired clinically.

Design specifications

We obtained preoperative 3-tesla MRI data, including T1-weighted spoiled gradient-echo volumetric and fluid-attenuated inversion recovery images (Nakai et al., 2017; Sakakura et al., 2022). The FreeSurfer software package was used to reconstruct the MRI surface image of patients aged two and above (http://surfer.nmr.mgh.harvard.edu; Desikan et al., 2006; Ghosh et al., 2010). In cases where the software failed to detect the pial surface accurately due to insufficient cerebral myelination, a board-certified neurosurgeon (K.S.) manually delineated the pial surface using the Control Point function (https://surfer.nmr.mgh.harvard.edu/fswiki/FsTutorial/ControlPoints_freeview/; Deoni et al., 2015; Croteau-Chonka et al., 2016; Remer et al., 2017; Sakakura et al., 2022). For patients younger than two, we used the Infant FreeSurfer software package to reconstruct the surface image (https://surfer.nmr.mgh.harvard.edu/fswiki/infantFS; Zöllei et al., 2020; Sakakura et al., 2022). We displayed electrode sites on the pial surface of the brain using preoperative MRI and a post-implant CT image (Stolk et al., 2018; Sakakura et al., 2022). Two board-certified neurosurgeons (K.S. and N.K.) visually assessed intraoperative photographs to confirm the spatial accuracy of electrode locations co-registered to the MRI surface image (Pieters et al., 2013). In order to pool sites from all patients, it was necessary to normalize the electrode locations to the standardized FSaverage brain surface (http://surfer.nmr.mgh.harvard.edu). In a previous iEEG study of 32 patients, we found that the mean coregistration error ranged below 0.4 mm, and there was no significant correlation between patient age and the severity of coregistration error (Sakakura et al., 2022).

Behavioral performance measures

We did not use behavioral performance measures in the present study.

Acquisition

Imaging type(s)

Structural

Field strength

3 Tesla

Sequence & imaging parameters

T1-weighted spoiled gradient-echo volumetric and fluid-attenuated inversion recovery images

Area of acquisition

Whole brain

Diffusion MRI ☒ Used ☐ Not used

Parameters

We delineated white matter DWI streamlines using open-source data from 1,065 healthy participants (http://brain.labsolver.org/diffusion-mri-templates/hcp-842-hcp-1021; Yeh et al., 2018), as previously reported (Mitsuhashi et al., 2021, 2022; Sonoda et al., 2021; Kitazawa et al., 2023; Ono et al., 2023). Our group validated the use of open-source DWI data by demonstrating that the inferred velocity of neural propagations induced by single-pulse electrical stimulation was similar whether using open-source or individual patient DWI data (Sonoda et al., 2021). In other words, the current study did not use individual patients' diffusion MRI.

Preprocessing

Preprocessing software

DSI Studio

Normalization

We delineated white matter DWI streamlines using open-source data from 1,065 healthy participants (http://brain.labsolver.org/diffusion-mri-templates/hcp-842-hcp-1021; Yeh et al., 2018), as previously reported (Mitsuhashi et al., 2021, 2022; Sonoda et al., 2021; Kitazawa et al., 2023; Ono et al., 2023). Our group validated the use of open-source DWI data by demonstrating that the inferred velocity of neural propagations induced by single-pulse electrical stimulation was similar whether using open-source or individual patient DWI data (Sonoda et al., 2021).

Normalization template

Open-source data from 1,065 healthy participants (http://brain.labsolver.org/diffusion-mri-templates/hcp-842-hcp-1021; Yeh et al., 2018)

Noise and artifact removal

We placed seeds (4-mm radius) at cortical mesh points demonstrating significant developmental enhancement (or reduction) of nested HFO-based neural communications (as rated by MI≥80 Hz & 0.5-1 Hz). Using DSI Studio (http://dsi-studio.labsolver.org/), we visualized DWI streamlines directly connecting the mesh points within Montreal Neurological Institute standard space. For fiber tracking, we utilized the following parameters: a quantitative anisotropy threshold of 0.05, a maximum turning angle of 70°, and a streamline length of 20 to 250 mm. In this investigation, we exclusively visualized DWI streamlines with at least 50% of their coordinates in one of the following white matter tracts: arcuate fasciculus, cingulum, corpus callosum, extreme capsule, frontal aslant tract, inferior fronto-occipital fasciculus, inferior longitudinal fasciculus, middle longitudinal fasciculus, superior longitudinal fasciculus, uncinate fasciculus, or vertical occipital fasciculus, as defined

in DSI Studio (as previously performed in Sonoda et al. 2021). We excluded streamlines involving the brainstem, basal ganglia, thalamus, or cerebrospinal fluid space.

Volume censoring

We used the open-source data from 1,065 healthy participants (http://brain.labsolver.org/diffusion-mri-templates/hcp-842-hcp-1021; Yeh et al., 2018) to generate a dynamic tractography atlas.

Statistical modeling & inference

Model type and settings

Statistical analysis to confirm physiological enhancement of MI and HFO in the occipital lobe. We used mixed model analysis with electrode location in the occipital lobe (yes = 1) as the fixed effect predictor variable and either MI≥80 Hz & 0.5-1 Hz, MI≥80 Hz & 3-4 Hz, HFOSTE ≥80 Hz, HFOSLL ≥80 Hz, HFOHIL ≥80 Hz, or HFOMNI ≥80 Hz as the dependent variable. The random effect factors included intercept and patient. We considered an FDR-corrected p-value of less than 0.05 as significant, for comparisons of six iEEG measures. We reported the mixed model effect and 95% confidence interval (95% CI) to highlight the impact of topography on normative iEEG biomarker measures. All statistical analyses were performed using Matlab R2020a (MathWorks Inc., Natick, MA).
Statistical analysis to visualize the developmental slope of cortical MI and HFO at given mesh points. We presented iEEG biomarker measures at each nonepileptic electrode site on a standardized brain surface image using FreeSurfer and interpolation within 10 mm from the electrode center at the individual patient level (Sakakura et al., 2022, Ono et al., 2023). Using linear and nonlinear univariate regression models at cortical surface mesh points consisting of 20 neighboring FreeSurfer vertex finite elements (Desikan et al., 2006; Sakakura et al., 2022), we then determined the developmental slope of the iEEG biomarker, at the whole brain level. We used age, square root of age (√age), and log10 age as an independent variable, and MI≥80 Hz & 0.5-1 Hz, MI≥80 Hz & 3-4 Hz, HFOSTE ≥80 Hz, HFOSLL ≥80 Hz, HFOHIL ≥80 Hz, or HFOMNI ≥80 Hz as dependent variables. We evaluated the goodness of fit of each regression model using Akaike Information Criterion (AIC). A biomarker value was considered to increase with age if the regression slope was significantly greater than zero. We identified the mesh points where the developmental change (enhancement or diminution) of a given iEEG biomarker measure survived an FDR correction (for 18 comparisons: six iEEG measures × three types of age measures) in the resulting normative atlas. We also created video atlases, each displaying MI≥80 Hz & 0.5-1 Hz, MI≥80 Hz & 3-4 Hz, or HFOHIL ≥80 Hz values predicted by a given regression model at cortical mesh points (Figure 3; Videos S1-S3).
Statistical analysis to determine the independent effects of development on cortical MI and HFO in each lobe. We used mixed model analysis to determine the lobe where a developmental change of a given iEEG biomarker remained significant, after controlling for the independent effects of patient demographics (age and sex) and epilepsy-related variables (SOZ location, MRI lesion, and number of oral antiseizure medications). The aim was to account for potential confounders that could affect MI and HFO measures.
To assess the developmental change of MI at each lobe, we utilized the MATLAB fitlme command (https://www.mathworks.com/help/stats/fitlme.html) to fit a mixed model specified by the following formula: 'MI ~ 1 + age + sex + SOZ + MRI + hemisphere + number of antiseizure medications + (1|patient)'. Here, the dependent variable was MI at a given analysis mesh point, and the fixed effect predictors included [1] age at surgery (e.g., √year), [2] sex (female = 1), [3] presence of SOZ in a given lobe (yes = 1), [4] presence of MRI-visible structural lesion (yes = 1), [5] sampled hemisphere (left = 1), and [6] number of oral antiseizure medications taken immediately before the initiation of iEEG recording. We considered a larger number of antiseizure medications as a surrogate of a more severe seizure-related cognitive burden since polytherapy is associated with more disabling seizures and seizure-related cognitive impairment (Kwan and Brodie, 2001; Kuroda et al., 2021). We employed this approach since no single neuropsychological assessment can quantify the severity of cognitive impairment across all age ranges. Our random effect factors included the intercept and patient. We deemed an FDR-corrected two-sided p-value of 0.05 (for 18 comparisons: six iEEG measures × three types of age measures) as the significance threshold.

Effect(s) tested

Phase-amplitude coupling rated by modulation index (MI) as well as high-frequency oscillation (HFO) event occurrence rate.

Specify type of analysis: ☐ Whole brain ☐ ROI-based ☒ Both

Anatomical location(s)

We performed the whole brain level analysis as well as ROI-based analysis, in which we determined whether the occipital lobe had a higher modulation index (MI) and high-frequency oscillation (HFO) event occurrence rate.

Statistic type for inference
(See Eklund et al. 2016)

We performed a statistical analysis to visualize the developmental slope of cortical MI and HFO at given cortical surface mesh points. Thereby, mesh points consisted of 20 neighboring FreeSurfer vertex finite elements (Desikan et al., 2006; Sakakura et al., 2022).

Correction

FDR

Models & analysis

n/a	Involved in the study
☐	☒ Functional and/or effective connectivity
☒	☐ Graph analysis
☐	☒ Multivariate modeling or predictive analysis

Functional and/or effective connectivity

Statistical analysis: white matter tracts between cortices showing developmental MI co-growth. We visualized white matter tracts directly connecting cortical mesh points that showed significant developmental co-growth (or co-reduction) of MI≥80 Hz & 0.5-1 Hz. To this end, we used the regression slope of MI≥80 Hz & 0.5-1 Hz as a function of √age at each cortical mesh point, as computed in a regression analysis mentioned above. We declared that the developmental enhancement (or reduction) of nested HFO-based neural communications took place between two cortical mesh points only if [1] two distinct mesh

points showed significantly positive (or negative) regression slopes, and [2] these mesh points were accompanied by direct tractography streamlines on diffusion-weighted imaging (DWI) analysis (Kitazawa et al., 2023; Ono et al., 2023).
We delineated white matter DWI streamlines using open-source data from 1,065 healthy participants (http://brain.labsolver.org/diffusion-mri-templates/hcp-842-hcp-1021; Yeh et al., 2018), as previously reported (Mitsuhashi et al., 2021, 2022; Sonoda et al., 2021; Kitazawa et al., 2023; Ono et al., 2023). Our group validated the use of open-source DWI data by demonstrating that the inferred velocity of neural propagations induced by single-pulse electrical stimulation was similar whether using open-source or individual patient DWI data (Sonoda et al., 2021). We placed seeds (4-mm radius) at cortical mesh points demonstrating significant developmental enhancement (or reduction) of nested HFO-based neural communications (as rated by MI≥80 Hz & 0.5-1 Hz). Using DSI Studio (http://dsi-studio.labsolver.org/), we visualized DWI streamlines directly connecting the mesh points within Montreal Neurological Institute standard space. For fiber tracking, we utilized the following parameters: a quantitative anisotropy threshold of 0.05, a maximum turning angle of 70°, and a streamline length of 20 to 250 mm. In this investigation, we exclusively visualized DWI streamlines with at least 50% of their coordinates in one of the following white matter tracts: arcuate fasciculus, cingulum, corpus callosum, extreme capsule, frontal aslant tract, inferior fronto-occipital fasciculus, inferior longitudinal fasciculus, middle longitudinal fasciculus, superior longitudinal fasciculus, uncinate fasciculus, or vertical occipital fasciculus, as defined in DSI Studio (as previously performed in Sonoda et al. 2021). We excluded streamlines involving the brainstem, basal ganglia, thalamus, or cerebrospinal fluid space.
The resulting dynamic tractography video atlases highlighted the intensity of MI≥80 Hz & 0.5-1 Hz developmental co-growth (or co-reduction) via given tractography streamlines for every 0.1 years; thereby, the intensity was defined as (√|regression slope at a mesh point| × √|regression slope at another mesh point|), at a given streamline connecting a pair of mesh points (Figure 5).

Multivariate modeling and predictive analysis

Statistical analysis to confirm physiological enhancement of MI and HFO in the occipital lobe during young childhood
In a previous study, we created normative atlases of MI and HFO based on data from 47 patients aged between 4 and 19 years, which revealed a general enhancement of these measures in the occipital lobe (Kuroda et al., 2021). In the current study, we aimed to replicate this finding in a cohort of 14 children aged between 1.0 and 3.9 years and 100 individuals aged 4 years or older. We used mixed model analysis with electrode location in the occipital lobe (yes = 1) as the fixed effect predictor variable and either MI≥80 Hz & 0.5-1 Hz, MI≥80 Hz & 3-4 Hz, HFOSTE ≥80 Hz, HFOSLL ≥80 Hz, HFOHIL ≥80 Hz, or HFOMNI ≥80 Hz as the dependent variable. The random effect factors included intercept and patient. We considered an FDR-corrected p-value of less than 0.05 as significant, for comparisons of six iEEG measures. We reported the mixed model effect and 95% confidence interval (95% CI) to highlight the impact of topography on normative iEEG biomarker measures. All statistical analyses were performed using Matlab R2020a (MathWorks Inc., Natick, MA).

Statistical analysis to visualize the developmental slope of cortical MI and HFO at given mesh points
We presented iEEG biomarker measures at each nonepileptic electrode site on a standardized brain surface image using FreeSurfer and interpolation within 10 mm from the electrode center at the individual patient level (Sakakura et al., 2022, Ono et al., 2023). Using linear and nonlinear univariate regression models at cortical surface mesh points consisting of 20 neighboring FreeSurfer vertex finite elements (Desikan et al., 2006; Sakakura et al., 2022), we then determined the developmental slope of the iEEG biomarker, at the whole brain level. We used age, square root of age (√age), and log10 age as an independent variable, and MI≥80 Hz & 0.5-1 Hz, MI≥80 Hz & 3-4 Hz, HFOSTE ≥80 Hz, HFOSLL ≥80 Hz, HFOHIL ≥80 Hz, or HFOMNI ≥80 Hz as dependent variables. We evaluated the goodness of fit of each regression model using Akaike Information Criterion (AIC). A biomarker value was considered to increase with age if the regression slope was significantly greater than zero. We identified the mesh points where the developmental change (enhancement or diminution) of a given iEEG biomarker measure survived an FDR correction (for 18 comparisons: six iEEG measures × three types of age measures) in the resulting normative atlas. We also created video atlases, each displaying MI≥80 Hz & 0.5-1 Hz, MI≥80 Hz & 3-4 Hz, or HFOHIL ≥80 Hz values predicted by a given regression model at cortical mesh points (Figure 3; Videos S1-S3).

Statistical analysis to determine the independent effects of development on cortical MI and HFO in each lobe
We used mixed model analysis33, 47 to determine the lobe where a developmental change of a given iEEG biomarker remained significant, after controlling for the independent effects of patient demographics (age and sex) and epilepsy-related variables (SOZ location, MRI lesion, and number of oral antiseizure medications). The aim was to account for potential confounders that could affect MI and HFO measures.
To assess the developmental change of MI at each lobe, we utilized the MATLAB fitlme command (https://www.mathworks.com/help/stats/fitlme.html) to fit a mixed model specified by the following formula: 'MI ~ 1 + age + sex + SOZ + MRI + hemisphere + number of antiseizure medications + (1|patient)'. Here, the dependent variable was MI at a given analysis mesh point, and the fixed effect predictors included [1] age at surgery (e.g., √year), [2] sex (female = 1), [3] presence of SOZ in a given lobe (yes = 1), [4] presence of MRI-visible structural lesion (yes = 1), [5] sampled hemisphere (left = 1), and [6] number of oral antiseizure medications taken immediately before the initiation of iEEG recording. We considered a larger number of antiseizure medications as a surrogate of a more severe seizure-related cognitive burden since polytherapy is associated with more disabling seizures and seizure-related cognitive impairment (Kwan and Brodie, 2001; Kuroda et al., 2021). We employed this approach since no single neuropsychological assessment can quantify the severity of cognitive impairment across all age ranges. Our random effect factors included the intercept and patient. We deemed an FDR-corrected p-value of 0.05 (for 18 comparisons: six iEEG measures × three types of age measures) as the significance threshold.

Statistical analysis: white matter tracts between cortices showing developmental MI co-growth
We visualized white matter tracts directly connecting cortical mesh points that showed significant

developmental co-growth (or co-reduction) of MI≥80 Hz & 0.5-1 Hz. To this end, we used the regression slope of MI≥80 Hz & 0.5-1 Hz as a function of √age at each cortical mesh point, as computed in a regression analysis mentioned above. We declared that the developmental enhancement (or reduction) of nested HFO-based neural communications took place between two cortical mesh points only if [1] two distinct mesh points showed significantly positive (or negative) regression slopes, and [2] these mesh points were accompanied by direct tractography streamlines on diffusion-weighted imaging (DWI) analysis (Kitazawa et al., 2023; Ono et al., 2023).
We delineated white matter DWI streamlines using open-source data from 1,065 healthy participants (http://brain.labsolver.org/diffusion-mri-templates/hcp-842-hcp-1021; Yeh et al., 2018), as previously reported (Mitsuhashi et al., 2021, 2022; Sonoda et al., 2021; Kitazawa et al., 2023; Ono et al., 2023). Our group validated the use of open-source DWI data by demonstrating that the inferred velocity of neural propagations induced by single-pulse electrical stimulation was similar whether using open-source or individual patient DWI data (Sonoda et al., 2021). We placed seeds (4-mm radius) at cortical mesh points demonstrating significant developmental enhancement (or reduction) of nested HFO-based neural communications (as rated by MI≥80 Hz & 0.5-1 Hz). Using DSI Studio (http://dsi-studio.labsolver.org/), we visualized DWI streamlines directly connecting the mesh points within Montreal Neurological Institute standard space. For fiber tracking, we utilized the following parameters: a quantitative anisotropy threshold of 0.05, a maximum turning angle of 70°, and a streamline length of 20 to 250 mm. In this investigation, we exclusively visualized DWI streamlines with at least 50% of their coordinates in one of the following white matter tracts: arcuate fasciculus, cingulum, corpus callosum, extreme capsule, frontal aslant tract, inferior fronto-occipital fasciculus, inferior longitudinal fasciculus, middle longitudinal fasciculus, superior longitudinal fasciculus, uncinate fasciculus, or vertical occipital fasciculus, as defined in DSI Studio (as previously performed in Sonoda et al. 2021). We excluded streamlines involving the brainstem, basal ganglia, thalamus, or cerebrospinal fluid space.
The resulting dynamic tractography video atlases highlighted the intensity of MI≥80 Hz & 0.5-1 Hz developmental co-growth (or co-reduction) via given tractography streamlines for every 0.1 years; thereby, the intensity was defined as (√|regression slope at a mesh point| × √|regression slope at another mesh point|), at a given streamline connecting a pair of mesh points (Figure 5).
